# Supplementary material for: Effects of plant growth-promoting rhizobacteria on blueberry growth and rhizosphere soil microenvironment
Source: PeerJ. 2024 Feb 26;12:e16992. doi: 10.7717/peerj.16992 (PMC10903360; doi:10.7717/peerj.16992)
Supplement: Supplemental Information 1 [file peerj-12-16992-s001.docx]

[Appendix](javascript:;) Table 1 Classifications of identified PGPR strains

| PGPR strains | Strain (Accession number) | Phosphorus solubilizing capabilities (mg/L) | Auxin production capabilities (mg/L) |
| --- | --- | --- | --- |
| B1 | *Pseudomonas umsongensis*  (MW407039) | 1.029±0.049 | 14.974±2.118 |
| B2 | *Pseudomonas extremorientalis*  (MW407037) | 0.353±0.050 | 14.286±1.794 |
| B3 | *Pseudomonas koreensis* (MW407038) | 0.427±0.190 | 14.471±1.489 |
| B4 | *Pseudomonas reinekei* (MW407039) | 0.196±0.068 | 12.513±1.083 |
| B5 | *Pseudomonas* sp. (MW407036) | 0.642±0.039 | 20.053±6.642 |
| B6 | *Buttiauxella* sp. | 4.269±0.599 | 15.100±1.500 |
| B7 | *Buttiauxella brennerae* (MW407042) | 3.445±0.059 | 58.069±3.352 |
| B8 | *Buttiauxella* sp. (MW407041) | 4.076±0.211 | 58.016±2.476 |
| B9 | *Buttiauxella gaviniae* (MW407043) | 4.985±0.509 | 51.111±3.742 |
