# Supplementary material for: Effects of plant growth-promoting rhizobacteria on blueberry growth and rhizosphere soil microenvironment
Source: PeerJ. 2024 Feb 26;12:e16992. doi: 10.7717/peerj.16992 (PMC10903360; doi:10.7717/peerj.16992)
Supplement: Supplemental Information 2 — Phosphorus: Phosphorus- solubilizing capacity of strains used for root irrigation, Auxin: Auxin production capacity of strains used for root irrigation,OCC: organic carbon content, TNC: total nitrogen content, TPHC: total phosphorous content, TPOC: total potassium content, HNC: hydrolysable nitrogen content, APHC: available phosphorous content, and APOC: available potassium content * p < 0.05; ** p < 0.01 [file peerj-12-16992-s002.docx]

[Appendix](javascript:;) Table 2

Kendall's tau correlation analysis of phosphorus solubilizing ability and auxin production ability of PGPR strains with rhizosphere soil microbial diversity, soil element content and plant growth status

| Capacity of strains | Acidobacteriota | Actinomycetota | Bacteroidota | Pseudomonadota | Verrucomicrobiota | Ascomycota | Basidiomycota | Mucoromycota |
| --- | --- | --- | --- | --- | --- | --- | --- | --- |
| Phosphorus | 0.613^**^ | 0.390^**^ | 0.765^**^ | -0.187 | -0.561^**^ | 0.424^**^ | -0.429^**^ | -0.220 |
| Auxin | 0.501^**^ | 0.349^**^ | 0.653^**^ | -0.321^*^ | -0.465^**^ | 0.421^**^ | -0.576^**^ | -0.195 |
|  | OCC | TNC | HNC | TPHC | APHC | TPOC | APOC | - |
| Phosphorus | 0.313^*^ | 0.266^*^ | 0.102 | 0.140 | -0.049 | 0.069 | -0.097 | - |
| Auxin | 0.228 | 0.143 | -0.024 | 0.218 | 0.016 | 0.033 | -0.211 | - |
|  | Branch Number | Leaf Number | Chl | Primary Root Length | Plant Height | - | - | - |
| Phosphorus | 0.219 | 0.276^*^ | 0.388^**^ | 0.289^*^ | 0.134 | - | - | - |
| Auxin | 0.096 | 0.204 | 0.159 | 0.073 | 0.028 | - | - | - |

Phosphorus: Phosphorus- solubilizing capacity of strains used for root irrigation, Auxin: Auxin production capacity of strains used for root irrigation，OCC: organic carbon content, TNC: total nitrogen content, TPHC: total phosphorous content, TPOC: total potassium content, HNC: hydrolysable nitrogen content, APHC: available phosphorous content, and APOC: available potassium content

* p < 0.05; ** p < 0.01
