# Supplementary material for: Effects of plant growth-promoting rhizobacteria on blueberry growth and rhizosphere soil microenvironment
Source: PeerJ. 2024 Feb 26;12:e16992. doi: 10.7717/peerj.16992 (PMC10903360; doi:10.7717/peerj.16992)
Supplement: Supplemental Information 3 — OCC: organic carbon content, TNC: total nitrogen content, TPHC: total phosphorous content, TPOC: total potassium content, HNC: hydrolysable nitrogen content, APHC: available phosphorous content, and APOC: available potassium content * p < 0.05; ** p < 0.01 [file peerj-12-16992-s003.docx]

[Appendix](javascript:;) Table 3 Kendall's tau correlation analysis of rhizosphere soil microbial diversity with plant growth status and soil element contents, Kendall's tau correlation analysis of soil element contents with plant growth status

|  | Branch Number | Leaf Number | Chl | Primary Root Length | Plant Height | OCC | TNC | HNC | TPHC | APHC | TPOC | APOC |
| --- | --- | --- | --- | --- | --- | --- | --- | --- | --- | --- | --- | --- |
| Acidobacteriota | 0.198 | 0.233 | 0.396^**^ | 0.427^**^ | 0.280^*^ | 0.290^*^ | 0.254 | 0.168 | 0.198 | 0.002 | 0.065 | -0.167 |
| Actinomycetota | 0.338^**^ | 0.153 | 0.109 | 0.023 | 0.114 | 0.000 | 0.023 | 0.100 | 0.324^*^ | -0.158 | 0.434^**^ | -0.016 |
| Bacteroidota | 0.285^*^ | 0.160 | 0.256^*^ | 0.221 | 0.075 | 0.207 | 0.160 | 0.037 | 0.174 | -0.053 | 0.121 | -0.204 |
| Pseudomonadota | 0.150 | 0.103 | 0.069 | 0.206 | 0.264^*^ | -0.021 | 0.058 | 0.366^**^ | 0.201 | -0.173 | 0.424^**^ | 0.474^**^ |
| Verrucomicrobiota | -0.157 | -0.485^**^ | -0.567^**^ | -0.431^**^ | -0.383^**^ | -0.251 | -0.241 | -0.142 | -0.429^**^ | -0.213 | -0.275^*^ | 0.035 |
| Ascomycota | 0.313^*^ | 0.124 | 0.228 | 0.429^**^ | 0.159 | 0.203 | 0.120 | 0.266^*^ | 0.259^*^ | -0.088 | 0.108 | -0.191 |
| Basidiomycota | -0.359^**^ | -0.114 | -0.016 | -0.171 | -0.155 | -0.324^*^ | -0.194 | -0.016 | -0.128 | 0.180 | -0.035 | 0.228 |
| Mucoromycota | -0.123 | 0.005 | -0.190 | -0.323^*^ | -0.005 | 0.002 | 0.063 | -0.065 | -0.070 | -0.039 | 0.097 | 0.370^**^ |
| OCC | 0.211 | 0.359^**^ | 0.056 | 0.053 | 0.195 | - | - | - | - | - | - | - |
| TNC | 0.210 | 0.377^**^ | 0.037 | 0.053 | 0.171 | - | - | - | - | - | - | - |
| HNC | -0.148 | -0.148 | 0.230 | 0.427^**^ | 0.077 | - | - | - | - | - | - | - |
| TPHC | 0.201 | 0.303^*^ | 0.498^**^ | 0.440^**^ | 0.354^**^ | - | - | - | - | - | - | - |
| APHC | -0.279^*^ | 0.033 | 0.277^*^ | 0.123 | -0.046 | - | - | - | - | - | - | - |
| TPOC | 0.388^**^ | 0.329^*^ | 0.413^**^ | 0.338^**^ | 0.535^**^ | - | - | - | - | - | - | - |
| APOC | 0.092 | 0.152 | -0.145 | -0.060 | 0.044 | - | - | - | - | - | - | - |

OCC: organic carbon content, TNC: total nitrogen content, TPHC: total phosphorous content, TPOC: total potassium content, HNC: hydrolysable nitrogen content, APHC: available phosphorous content, and APOC: available potassium content

* p < 0.05; ** p < 0.01
