# Supplementary material for: Effects of plant growth-promoting rhizobacteria on blueberry growth and rhizosphere soil microenvironment
Source: PeerJ. 2024 Feb 26;12:e16992. doi: 10.7717/peerj.16992 (PMC10903360; doi:10.7717/peerj.16992)
Supplement: Supplemental Information 4 — Phosphorus: Phosphorus- solubilizing capacity of strains used for root irrigation, Auxin: Auxin production capacity of strains used for root irrigation,OCC: organic carbon content, TNC: total nitrogen content, TPHC: total phosphorous content, TPOC: total potassium content, HNC: hydrolysable nitrogen content, APHC: available phosphorous content, and APOC: available potassium content * p < 0.05; ** p < 0.01 [file peerj-12-16992-s004.docx]

[Appendix](javascript:;) Table 4 Spearman's rank correlation analysis of phosphorus solubilizing ability and auxin production ability of PGPR strains with rhizosphere soil microbial diversity, soil element content and plant growth status

| Capacity of strains | Acidobacteriota | Actinomycetota | Bacteroidota | Pseudomonadota | Verrucomicrobiota | Ascomycota | Basidiomycota | Mucoromycota |
| --- | --- | --- | --- | --- | --- | --- | --- | --- |
| Phosphorus | 0.825^**^ | 0.530^**^ | 0.916^**^ | -0.242 | -0.662^**^ | 0.677^**^ | -0.709^**^ | -0.518^**^ |
| Auxin | 0.692^**^ | 0.456^*^ | 0.863^**^ | -0.334 | -0.640^**^ | 0.613^**^ | -0.743^**^ | -0.402^*^ |
|  | OCC | TNC | HNC | TPHC | APHC | TPOC | APOC | - |
| Phosphorus | 0.448^*^ | 0.395^*^ | 0.082 | 0.245 | -0.083 | 0.137 | -0.128 | - |
| Auxin | 0.257 | 0.190 | -0.029 | 0.307 | 0.001 | 0.102 | -0.234 | - |
|  | Branch Number | Leaf Number | Chl | Primary Root Length | Plant Height | - | - | - |
| Phosphorus | 0.299 | 0.417^*^ | 0.496^**^ | 0.362^*^ | 0.153 | - | - | - |
| Auxin | 0.199 | 0.221 | 0.273 | 0.135 | 0.055 | - | - | - |

Phosphorus: Phosphorus- solubilizing capacity of strains used for root irrigation, Auxin: Auxin production capacity of strains used for root irrigation，OCC: organic carbon content, TNC: total nitrogen content, TPHC: total phosphorous content, TPOC: total potassium content, HNC: hydrolysable nitrogen content, APHC: available phosphorous content, and APOC: available potassium content

* p < 0.05; ** p < 0.01
