# Supplementary material for: Effects of plant growth-promoting rhizobacteria on blueberry growth and rhizosphere soil microenvironment
Source: PeerJ. 2024 Feb 26;12:e16992. doi: 10.7717/peerj.16992 (PMC10903360; doi:10.7717/peerj.16992)
Supplement: Supplemental Information 5 — OCC: organic carbon content, TNC: total nitrogen content, TPHC: total phosphorous content, TPOC: total potassium content, HNC: hydrolysable nitrogen content, APHC: available phosphorous content, and APOC: available potassium content * p < 0.05; ** p < 0.01 [file peerj-12-16992-s005.docx]

[Appendix](javascript:;) Table 5 Spearman's rank correlation analysis of rhizosphere soil microbial diversity with plant growth status and soil element contents, Spearman's rank correlation analysis of soil element contents with plant growth status

|  | Branch Number | Leaf Number | Chl | Primary Root Length | Plant Height | OCC | TNC | HNC | TPHC | APHC | TPOC | APOC |
| --- | --- | --- | --- | --- | --- | --- | --- | --- | --- | --- | --- | --- |
| Acidobacteriota | 0.257 | 0.339 | 0.571^**^ | 0.567^**^ | 0.425^*^ | 0.443^*^ | 0.373^*^ | 0.246 | 0.317 | 0.011 | 0.141 | -0.219 |
| Actinomycetota | 0.481^**^ | 0.228 | 0.240 | 0.109 | 0.166 | -0.023 | 0.045 | 0.201 | 0.419^*^ | -0.258 | 0.567^**^ | 0.061 |
| Bacteroidota | 0.405^*^ | 0.243 | 0.340 | 0.280 | 0.094 | 0.302 | 0.214 | 0.052 | 0.261 | -0.127 | 0.147 | -0.210 |
| Pseudomonadota | 0.235 | 0.156 | 0.108 | 0.196 | 0.415^*^ | 0.027 | 0.128 | 0.475^**^ | 0.342 | -0.231 | 0.580^**^ | 0.638^**^ |
| Verrucomicrobiota | -0.191 | -0.656^**^ | -0.734^**^ | -0.606^**^ | -0.414^*^ | -0.379^*^ | -0.337 | -0.187 | -0.582^**^ | -0.287 | -0.352 | 0.068 |
| Ascomycota | 0.453^*^ | 0.129 | 0.403^*^ | 0.607^**^ | 0.354 | 0.301 | 0.143 | 0.391^*^ | 0.361 | -0.164 | 0.193 | -0.285 |
| Basidiomycota | -0.534^**^ | -0.117 | -0.128 | -0.304 | -0.240 | -0.413^*^ | -0.250 | -0.060 | -0.170 | 0.278 | -0.049 | 0.228 |
| Mucoromycota | -0.160 | 0.043 | -0.290 | -0.415^*^ | -0.039 | -0.051 | 0.087 | -0.131 | -0.090 | -0.056 | 0.119 | 0.609^**^ |
| OCC | 0.356 | 0.533^**^ | 0.077 | 0.111 | 0.297 | - | - | - | - | - | - | - |
| TNC | 0.293 | 0.528^**^ | 0.030 | 0.073 | 0.262 | - | - | - | - | - | - | - |
| HNC | -0.114 | -0.209 | 0.368^*^ | 0.618^**^ | 0.153 | - | - | - | - | - | - | - |
| TPHC | 0.305 | 0.390^*^ | 0.678^**^ | 0.597^**^ | 0.510^**^ | - | - | - | - | - | - | - |
| APHC | -0.398^*^ | 0.030 | 0.384^*^ | 0.178 | -0.055 | - | - | - | - | - | - | - |
| TPOC | 0.586^**^ | 0.534^**^ | 0.565^**^ | 0.480^**^ | 0.690^**^ | - | - | - | - | - | - | - |
| APOC | 0.144 | 0.236 | -0.241 | -0.158 | 0.097 | - | - | - | - | - | - | - |

OCC: organic carbon content, TNC: total nitrogen content, TPHC: total phosphorous content, TPOC: total potassium content, HNC: hydrolysable nitrogen content, APHC: available phosphorous content, and APOC: available potassium content

* p < 0.05; ** p < 0.01
