# Supplementary material for: Effects of plant growth-promoting rhizobacteria on blueberry growth and rhizosphere soil microenvironment
Source: PeerJ. 2024 Feb 26;12:e16992. doi: 10.7717/peerj.16992 (PMC10903360; doi:10.7717/peerj.16992)
Supplement: Supplemental Information 6 [file peerj-12-16992-s006.docx]

[Appendix](javascript:;) Table 6 Eigenvector of five principal components of phosphorus solubilizing ability and auxin production ability of PGPR strains, rhizosphere microenvironment factors and plant growth status

| Factors | Principal component | | | | |
| --- | --- | --- | --- | --- | --- |
|  | 1 | 2 | 3 | 4 | 5 |
| OCC | 0.427 | 0.420 | 0.635 | 0.309 | -0.263 |
| TNC | 0.361 | 0.359 | 0.630 | 0.319 | -0.215 |
| HNC | 0.249 | 0.344 | -0.509 | -0.377 | -0.602 |
| TPHC | 0.421 | 0.616 | -0.514 | 0.053 | 0.104 |
| APHC | -0.006 | 0.015 | -0.694 | 0.446 | 0.123 |
| TPOC | 0.110 | 0.743 | -0.249 | -0.293 | 0.480 |
| APOC | -0.336 | 0.553 | 0.537 | 0.024 | -0.355 |
| Branch Number | 0.563 | 0.478 | 0.319 | -0.178 | 0.323 |
| Leaf Number | 0.441 | 0.450 | -0.608 | 0.259 | -0.259 |
| Chl | 0.464 | 0.673 | 0.092 | 0.189 | 0.231 |
| Primary Root Length | 0.561 | 0.388 | -0.544 | 0.293 | -0.003 |
| Plant Height | 0.538 | 0.192 | 0.331 | 0.483 | 0.421 |
| Phosphorus | 0.927 | -0.289 | 0.087 | -0.003 | -0.049 |
| Auxin | 0.794 | -0.263 | 0.160 | -0.103 | 0.283 |
| Acidobacteriota | 0.947 | -0.233 | 0.050 | 0.027 | -0.088 |
| Actinomycetota | 0.129 | 0.412 | 0.083 | -0.707 | 0.260 |
| Bacteroidota | 0.888 | -0.236 | 0.088 | -0.281 | -0.017 |
| Pseudomonadota | -0.365 | 0.884 | 0.094 | -0.192 | -0.158 |
| Verrucomicrobiota | -0.842 | -0.253 | 0.084 | -0.010 | 0.196 |
| Ascomycota | 0.906 | -0.184 | 0.009 | -0.211 | -0.240 |
| Basidiomycota | -0.944 | 0.127 | -0.127 | 0.158 | 0.127 |
| Mucoromycota | -0.682 | 0.587 | 0.213 | -0.006 | -0.080 |
| Accumulating contribution rate | 37.371% | 20.024 % | 14.397 % | 8.082 % | 7.110 % |

Note: Determinant factors were chosen when the absolute value is greater than or equal to 0.850．
