# Supplementary material for: Effects of plant growth-promoting rhizobacteria on blueberry growth and rhizosphere soil microenvironment
Source: PeerJ. 2024 Feb 26;12:e16992. doi: 10.7717/peerj.16992 (PMC10903360; doi:10.7717/peerj.16992)
Supplement: Supplemental Information 8 [file peerj-12-16992-s008.zip › 20240203raw data 20230921/translation-PRIncipal component analysis.doc]

GET
  FILE='C:\Users\nxygp\Desktop\raw data of plant physiology & soil samples & cor.sav'.
DATASET NAME data set 1 WINDOW=FRONT.
GET
  FILE='C:\Users\nxygp\Desktop\principal component analysis.sav'.
DATASET NAME data set 2 WINDOW=FRONT.
DATASET ACTIVATE data set 1.
FACTOR
  /VARIABLES ZOCC ZTNC ZHNC ZTPHC ZAPHC ZTPOC ZAPOC ZBranchnumber Zrootlength Zplantheight Zchl Zleafnumber ZPhosphorus Zauxin ZAcidobacteriota ZActinomycetota ZBacteroidota ZPseudomonadota ZVerrucomicrobiota ZAscomycota ZBasidiomycota ZMucoromycota
  /MISSING LISTWISE
  /ANALYSIS ZOCC ZTNC ZHNC ZTPHC ZAPHC ZTPOC ZAPOC ZBranchnumber Zrootlength Zplantheight Zchl Zleafnumber ZPhosphorus Zauxin ZAcidobacteriota ZActinomycetota ZBacteroidota ZPseudomonadota ZVerrucomicrobiota ZAscomycota ZBasidiomycota ZMucoromycota
  /PRINT INITIAL KMO EXTRACTION
  /CRITERIA MINEIGEN(1) ITERATE(25)
  /EXTRACTION PC
  /ROTATION NOROTATE
  /METHOD=CORRELATION.


factor analysis

Note	
Created output	21-SEP-2023 17:08:37	
annotation		
Input	data	C:\Users
xygp\Desktop\raw data of plant physiology & soil samples & cor.sav	
	A data set of activities	Data set 1	
	filter	<none>	
	weight	<none>	
	Split file	<none>	
	N lines in the working data file	32	
Missing value processing	The definition of missing	MISSING=EXCLUDE：User-defined missing values are treated as missing.	
	Use case	LISTWISE：The statistics are based on cases that do not contain missing values for any of the variables used.	
grammar	FACTOR
  /VARIABLES ZOCC ZTNC ZHNC ZTPHC ZAPHC ZTPOC ZAPOC ZBranchnumber Zrootlength Zplantheight Zchl Zleafnumber ZPhosphorus Zauxin ZAcidobacteriota ZActinomycetota ZBacteroidota ZPseudomonadota ZVerrucomicrobiota ZAscomycota ZBasidiomycota ZMucoromycota
  /MISSING LISTWISE
  /ANALYSIS ZOCC ZTNC ZHNC ZTPHC ZAPHC ZTPOC ZAPOC ZBranchnumber Zrootlength Zplantheight Zchl Zleafnumber ZPhosphorus Zauxin ZAcidobacteriota ZActinomycetota ZBacteroidota ZPseudomonadota ZVerrucomicrobiota ZAscomycota ZBasidiomycota ZMucoromycota
  /PRINT INITIAL KMO EXTRACTION
  /CRITERIA MINEIGEN(1) ITERATE(25)
  /EXTRACTION PC
  /ROTATION NOROTATE
  /METHOD=CORRELATION.	
resource	Processor time	00:00:00.05	
	Time spent	00:00:00.08	
	The maximum memory required	58024 (56.664K) byte	


[data set1] C:\Users\nxygp\Desktop\raw data of plant physiology & soil samples & cor.sav


KMO and Bartlett tests	
Sample the Kaiser-Meyer-Olkin measure of adequacy.	.510	
Sphericity test of Bartlett	Approximate chi-square	1306.014	
	df	231	
	Sig.	.000	


communality	
	initial	extract	
Zscore(OCC)	1.000	.927	
Zscore(TNC)	1.000	.804	
Zscore(HNC)	1.000	.944	
Zscore(TPHC)	1.000	.834	
Zscore(APHC)	1.000	.696	
Zscore(TPOC)	1.000	.943	
Zscore(APOC)	1.000	.834	
Zscore(Branchnumber)	1.000	.783	
Zscore(rootlength)	1.000	.900	
Zscore(plantheight)	1.000	.766	
Zscore(chl)	1.000	.847	
Zscore(leafnumber)	1.000	.846	
Zscore(Phosphorus)	1.000	.953	
Zscore(auxin)	1.000	.816	
Zscore(Acidobacteriota)	1.000	.963	
Zscore(Actinomycetota)	1.000	.761	
Zscore(Bacteroidota)	1.000	.931	
Zscore(Pseudomonadota)	1.000	.985	
Zscore(Verrucomicrobiota)	1.000	.818	
Zscore(Ascomycota)	1.000	.957	
Zscore(Basidiomycota)	1.000	.965	
Zscore(Mucoromycota)	1.000	.862	

Extraction method: principal component analysis	


Total Varian Explained	
Component	Initial Eigenvalues	Extraction Sums of Squared Loadings	
	Total	% of Variance 	% Cumulative 	Total	% of Variance 	% Cumulative 	
1	8.222	37.371	37.371	8.222	37.371	37.371	
2	4.405	20.024	57.395	4.405	20.024	57.395	
3	3.167	14.397	71.791	3.167	14.397	71.791	
4	1.778	8.082	79.874	1.778	8.082	79.874	
5	1.564	7.110	86.984	1.564	7.110	86.984	
6	.939	4.269	91.252				
7	.667	3.032	94.284				
8	.366	1.665	95.949				
9	.293	1.333	97.283				
10	.242	1.101	98.384				
11	.182	.829	99.213				
12	.094	.428	99.641				
13	.030	.135	99.776				
14	.018	.080	99.857				
15	.015	.066	99.923				
16	.010	.045	99.968				
17	.003	.015	99.983				
18	.002	.007	99.990				
19	.001	.005	99.995				
20	.001	.003	99.999				
21	.000	.001	100.000				
22	.000	.000	100.000				

Extraction method: principal component analysis	


Growth-share matrixa	
	Component	
	1	2	3	4	5	
Zscore(OCC)	.427	.420	.635	.309	-.263	
Zscore(TNC)	.361	.359	.630	.319	-.215	
Zscore(HNC)	.249	.344	-.509	-.377	-.602	
Zscore(TPHC)	.421	.616	-.514	.053	.104	
Zscore(APHC)	-.006	.015	-.694	.446	.123	
Zscore(TPOC)	.110	.743	-.249	-.293	.480	
Zscore(APOC)	-.336	.553	.537	.024	-.355	
Zscore(Branchnumber)	.563	.478	.319	-.178	.323	
Zscore(rootlength)	.441	.450	-.608	.259	-.259	
Zscore(plantheight)	.464	.673	.092	.189	.231	
Zscore(chl)	.561	.388	-.544	.293	-.003	
Zscore(leafnumber)	.538	.192	.331	.483	.421	
Zscore(Phosphorus)	.927	-.289	.087	-.003	-.049	
Zscore(auxin)	.794	-.263	.160	-.103	.283	
Zscore(Acidobacteriota)	.947	-.233	.050	.027	-.088	
Zscore(Actinomycetota)	.129	.412	.083	-.707	.260	
Zscore(Bacteroidota)	.888	-.236	.088	-.281	-.017	
Zscore(Pseudomonadota)	-.365	.884	.094	-.192	-.158	
Zscore(Verrucomicrobiota)	-.842	-.253	.084	-.010	.196	
Zscore(Ascomycota)	.906	-.184	.009	-.211	-.240	
Zscore(Basidiomycota)	-.944	.127	-.127	.158	.127	
Zscore(Mucoromycota)	-.682	.587	.213	-.006	-.080	

Extraction method: principal component analysis	
a. Five ingredients have been extracted	

DATASET ACTIVATE data set 1.

SAVE OUTFILE='C:\Users\nxygp\Desktop\raw data of plant physiology & soil samples & cor.sav'
  /COMPRESSED.
COMPUTE U1=A1 / sqrt(8.222).
EXECUTE.
COMPUTE U2=A2 / sqrt(4.405).
EXECUTE.
COMPUTE U3=A3 / sqrt(3.167).
EXECUTE.
COMPUTE U4=A4 / sqrt(1.778).
EXECUTE.
COMPUTE U5=A5 / sqrt(1.564).
EXECUTE.
DATASET ACTIVATE data set1.
DATASET CLOSE data set 2.
