# Supplementary material for: Effects of plant growth-promoting rhizobacteria on blueberry growth and rhizosphere soil microenvironment
Source: PeerJ. 2024 Feb 26;12:e16992. doi: 10.7717/peerj.16992 (PMC10903360; doi:10.7717/peerj.16992)
Supplement: Supplemental Information 8 [file peerj-12-16992-s008.zip › 20240203raw data 20230921/translation-different analysis-raw data of plant physiology & soil elements content -.doc]

GET
  FILE='C:\Users\nxygp\Documents\Tencent Files\403861433\FileRecv\MobileFile\raw data of plant physiology & soil samples & cor.sav'.
DATASET NAME data set 1 WINDOW=FRONT.
GLM OCC TNC HNC TPHC APHC TPOC APOC Branchnumber rootlength plantheight chl leafnumber BY 分组
  /METHOD=SSTYPE(3)
  /INTERCEPT=INCLUDE
  /POSTHOC=分组(DUNCAN LSD)
  /EMMEANS=TABLES(分组)
  /PRINT=DESCRIPTIVE
  /CRITERIA=ALPHA(.05)
  /DESIGN=分组.


general linear model

Note	
Created output	20-SEP-2023 07:25:55	
Annotation		
Input	Data	C:\Users
xygp\Documents\Tencent Files\403861433\FileRecv\MobileFile\raw data of plant physiology & soil samples & cor.sav	
	A data set of activities	 data set	
	N lines in the working data file	32	
Missing value processing	The definition of missing	User-defined missing values are treated as missing data.	
	Use case	The calculation of the statistics will be based on all cases with valid data that applies to all variables in the model.	
Grammar	GLM OCC TNC HNC TPHC APHC TPOC APOC Branchnumber rootlength plantheight chl leafnumber BY Group
  /METHOD=SSTYPE(3)
  /INTERCEPT=INCLUDE
  /POSTHOC=group(DUNCAN LSD)
  /EMMEANS=TABLES(group)
  /PRINT=DESCRIPTIVE
  /CRITERIA=ALPHA(.05)
  /DESIGN=group.	
Resource	Processor time	00:00:01.02	
	Time spent	00:00:01.22	


[data set 1] C:\Users\nxygp\Documents\Tencent Files\403861433\FileRecv\MobileFile\raw data of plant physiology & soil samples & cor.sav


Intersubjective factor	
	Value tag	N	
group	1	P5	3	
	2	P21	3	
	3	P22	3	
	4	P24	3	
	5	P26	3	
	6	P6	3	
	7	P7	3	
	8	P40	3	
	9	P42	3	
	10	CK	3	


Descriptive statistic	
	group	mean value	Standard deviation	N	
OCC	P5	534.000000	9.5393920	3	
	P21	510.666667	11.5902258	3	
	P22	529.333333	9.5043850	3	
	P24	580.333333	10.0166528	3	
	P26	541.333333	8.5049005	3	
	P6	569.666667	16.7431578	3	
	P7	465.000000	7.0000000	3	
	P40	584.666667	15.5670592	3	
	P42	557.000000	17.0000000	3	
	CK	433.000000	14.0000000	3	
	Total	530.500000	48.7829464	30	
TNC	P5	26.784000	3.2477635	3	
	P21	25.031333	1.0533496	3	
	P22	25.598000	1.5340596	3	
	P24	28.502333	2.1754416	3	
	P26	26.996000	.4280140	3	
	P6	28.188667	1.9189686	3	
	P7	22.559333	.9922683	3	
	P40	29.175333	2.7551873	3	
	P42	27.341000	1.0700575	3	
	CK	21.331667	.8797126	3	
	Total	26.150767	2.8967194	30	
HNC	P5	.780000	.0754983	3	
	P21	.860000	.0400000	3	
	P22	.940000	.0529150	3	
	P24	.685000	.0626498	3	
	P26	.638000	.0310483	3	
	P6	1.056667	.0513160	3	
	P7	.923333	.0680686	3	
	P40	.585333	.0174738	3	
	P42	.619333	.0380044	3	
	CK	.573333	.0750555	3	
	Total	.766100	.1709582	30	
TPHC	P5	330.000000	20.0000000	3	
	P21	345.000000	35.0000000	3	
	P22	445.000000	25.0000000	3	
	P24	260.000000	20.0000000	3	
	P26	255.000000	45.0000000	3	
	P6	300.000000	10.0000000	3	
	P7	350.000000	30.0000000	3	
	P40	325.000000	35.0000000	3	
	P42	320.000000	30.0000000	3	
	CK	226.000000	8.1853528	3	
	Total	315.600000	63.8319993	30	
APHC	P5	37.066667	.4041452	3	
	P21	36.533333	.2516611	3	
	P22	43.500000	2.7000000	3	
	P24	33.700000	.5000000	3	
	P26	37.283333	5.0000833	3	
	P6	37.350000	1.0500000	3	
	P7	38.750000	4.6500000	3	
	P40	38.250000	2.8500000	3	
	P42	37.300000	2.9000000	3	
	CK	40.800000	1.5000000	3	
	Total	38.053333	3.3907820	30	
TPOC	P5	2.423333	.2040425	3	
	P21	4.560000	.2505993	3	
	P22	3.110000	.1014889	3	
	P24	1.553333	.0503322	3	
	P26	1.320000	.1400000	3	
	P6	1.420000	.2400000	3	
	P7	1.890000	.2007486	3	
	P40	1.995000	.1003743	3	
	P42	2.548333	.1500278	3	
	CK	1.193333	.0862168	3	
	Total	2.201333	1.0019584	30	
APOC	P5	.454500	.0185000	3	
	P21	.337533	.0705000	3	
	P22	.351000	.1040000	3	
	P24	.469500	.0135000	3	
	P26	.408500	.0215000	3	
	P6	.333000	.0380000	3	
	P7	.224000	.0070000	3	
	P40	.296500	.0155000	3	
	P42	.280000	.0100000	3	
	CK	.230000	.0200000	3	
	Total	.338453	.0900827	30	
Branchnumber	P5	3.522233	.0693755	3	
	P21	3.862200	.0107014	3	
	P22	3.460000	.0100000	3	
	P24	3.903000	.1001349	3	
	P26	3.000000	.1000000	3	
	P6	3.206667	.0152753	3	
	P7	3.550000	.0100000	3	
	P40	3.640000	.0200000	3	
	P42	4.400000	.1732051	3	
	CK	2.580000	.1000000	3	
	Total	3.512410	.4933232	30	
rootlength	P5	9.690000	.0100000	3	
	P21	10.250000	.0500000	3	
	P22	13.300000	.2000000	3	
	P24	9.860000	.0600000	3	
	P26	8.380000	.0200000	3	
	P6	11.920000	.0200000	3	
	P7	10.440000	.2088061	3	
	P40	9.800000	.1000000	3	
	P42	11.030000	.0300000	3	
	CK	9.481667	.0851959	3	
	Total	10.415167	1.3374335	30	
plantheight	P5	13.7633	.25106	3	
	P21	16.8400	.04000	3	
	P22	16.3800	.08000	3	
	P24	15.7300	.03000	3	
	P26	14.1600	.16000	3	
	P6	15.0700	.07000	3	
	P7	14.1467	.16803	3	
	P40	16.1400	.14000	3	
	P42	16.2000	.20000	3	
	CK	12.9267	.08083	3	
	Total	15.1357	1.27751	30	
chl	P5	1.050000	.0500000	3	
	P21	1.220000	.2200000	3	
	P22	1.730000	.0300000	3	
	P24	.735667	.0350190	3	
	P26	.845000	.0050000	3	
	P6	1.531333	.0102632	3	
	P7	1.029000	.0010000	3	
	P40	1.180000	.0800000	3	
	P42	1.560000	.0600000	3	
	CK	.981333	.0015275	3	
	Total	1.186233	.3220323	30	
leafnumber	P5	30.940000	.0529150	3	
	P21	28.500000	.5000000	3	
	P22	31.300000	.3000000	3	
	P24	28.500000	.5000000	3	
	P26	27.200000	.2000000	3	
	P6	28.500000	.5000000	3	
	P7	24.666667	.1527525	3	
	P40	40.100000	.1000000	3	
	P42	35.070000	.0700000	3	
	CK	27.000000	.2000000	3	
	Total	30.177667	4.3467940	30	


Multivariate testa	
domino offect	Value	F	hypothesis df	error df	Sig.	
intercept	Track of Pillai 	1.000	221443.424b	12.000	9.000	.000	
	Lambda of Wilks  	.000	221443.424b	12.000	9.000	.000	
	Track of Hotelling 	295257.899	221443.424b	12.000	9.000	.000	
	Maximal root of Roy 	295257.899	221443.424b	12.000	9.000	.000	
group	Track of Pillai 	8.117	13.030	108.000	153.000	.000	
	Lambda of Wilks  	.000	106.711	108.000	78.042	.000	
	Track of Hotelling 	4151.967	277.652	108.000	65.000	.000	
	Maximal root of Roy 	2638.334	3737.640c	12.000	17.000	.000	

a. Design: Intercept + grouping	
b. Exact statistic	
c. This statistic is an upper bound on F, and it produces a lower bound on the level of significance.	


Examination of intersubjective effects	
ROOT	dependent variable	Sum of squares of type III	df	mean square	F	Sig.	
calibration model
	OCC	65923.500a	9	7324.833	47.410	.000	
	TNC	177.131b	9	19.681	5.945	.000	
	HNC	.788c	9	.088	29.552	.000	
	TPHC	102427.200d	9	11380.800	14.467	.000	
	APHC	184.875e	9	20.542	2.766	.028	
	TPOC	28.564f	9	3.174	115.502	.000	
	APOC	.197g	9	.022	11.535	.000	
	Branchnumber	6.926h	9	.770	116.977	.000	
	rootlength	51.656i	9	5.740	527.731	.000	
	plantheight	46.936j	9	5.215	264.993	.000	
	chl	2.881k	9	.320	50.685	.000	
	leafnumber	546.022l	9	60.669	631.290	.000	
intercept	OCC	8442907.500	1	8442907.500	54646.650	.000	
	TNC	20515.878	1	20515.878	6197.475	.000	
	HNC	17.607	1	17.607	5940.644	.000	
	TPHC	2988100.800	1	2988100.800	3798.272	.000	
	APHC	43441.685	1	43441.685	5848.763	.000	
	TPOC	145.376	1	145.376	5290.570	.000	
	APOC	3.437	1	3.437	1808.100	.000	
	Branchnumber	370.111	1	370.111	56258.246	.000	
	rootlength	3254.271	1	3254.271	299220.372	.000	
	plantheight	6872.652	1	6872.652	349220.130	.000	
	chl	42.214	1	42.214	6683.737	.000	
	leafnumber	27320.747	1	27320.747	284285.113	.000	
GROUP	OCC	65923.500	9	7324.833	47.410	.000	
	TNC	177.131	9	19.681	5.945	.000	
	HNC	.788	9	.088	29.552	.000	
	TPHC	102427.200	9	11380.800	14.467	.000	
	APHC	184.875	9	20.542	2.766	.028	
	TPOC	28.564	9	3.174	115.502	.000	
	APOC	.197	9	.022	11.535	.000	
	Branchnumber	6.926	9	.770	116.977	.000	
	rootlength	51.656	9	5.740	527.731	.000	
	plantheight	46.936	9	5.215	264.993	.000	
	chl	2.881	9	.320	50.685	.000	
	leafnumber	546.022	9	60.669	631.290	.000	
Error	OCC	3090.000	20	154.500			
	TNC	66.207	20	3.310			
	HNC	.059	20	.003			
	TPHC	15734.000	20	786.700			
	APHC	148.550	20	7.428			
	TPOC	.550	20	.027			
	APOC	.038	20	.002			
	Branchnumber	.132	20	.007			
	rootlength	.218	20	.011			
	plantheight	.394	20	.020			
	chl	.126	20	.006			
	leafnumber	1.922	20	.096			
Total	OCC	8511921.000	30				
	TNC	20759.216	30				
	HNC	18.455	30				
	TPHC	3106262.000	30				
	APHC	43775.110	30				
	TPOC	174.490	30				
	APOC	3.672	30				
	Branchnumber	377.168	30				
	rootlength	3306.144	30				
	plantheight	6919.981	30				
	chl	45.222	30				
	leafnumber	27868.691	30				
Corrected total	OCC	69013.500	29				
	TNC	243.339	29				
	HNC	.848	29				
	TPHC	118161.200	29				
	APHC	333.425	29				
	TPOC	29.114	29				
	APOC	.235	29				
	Branchnumber	7.058	29				
	rootlength	51.873	29				
	plantheight	47.329	29				
	chl	3.007	29				
	leafnumber	547.944	29				

a. R2 = .955（adjustment R2 = .935）	
b.  R2 = .728（adjustment R2  = .605）	
c.  R2 = .930（adjustment R2  = .899）	
d.  R2 = .867（adjustment R2 = .807）	
e.  R2 = .554（adjustment R2  = .354）	
f.  R2 = .981（adjustment R2  = .973）	
g.  R2 = .838（adjustment R2  = .766）	
h.  R2 = .981（adjustment R2  = .973）	
i. R2 = .996（adjustment R2 = .994）	
j.  R2 = .992（adjustment R2 = .988）	
k.  R2= .958（adjustment R2 = .939）	
l. R2= .996（adjustment R2 = .995）	


Estimated marginal mean


Group	
dependent variable	Group	Mean	Standard error	95% confidence interval	
				lower level	Up level	
OCC	P5	534.000	7.176	519.030	548.970	
	P21	510.667	7.176	495.697	525.636	
	P22	529.333	7.176	514.364	544.303	
	P24	580.333	7.176	565.364	595.303	
	P26	541.333	7.176	526.364	556.303	
	P6	569.667	7.176	554.697	584.636	
	P7	465.000	7.176	450.030	479.970	
	P40	584.667	7.176	569.697	599.636	
	P42	557.000	7.176	542.030	571.970	
	CK	433.000	7.176	418.030	447.970	
TNC	P5	26.784	1.050	24.593	28.975	
	P21	25.031	1.050	22.840	27.223	
	P22	25.598	1.050	23.407	27.789	
	P24	28.502	1.050	26.311	30.694	
	P26	26.996	1.050	24.805	29.187	
	P6	28.189	1.050	25.997	30.380	
	P7	22.559	1.050	20.368	24.751	
	P40	29.175	1.050	26.984	31.367	
	P42	27.341	1.050	25.150	29.532	
	CK	21.332	1.050	19.140	23.523	
HNC	P5	.780	.031	.714	.846	
	P21	.860	.031	.794	.926	
	P22	.940	.031	.874	1.006	
	P24	.685	.031	.619	.751	
	P26	.638	.031	.572	.704	
	P6	1.057	.031	.991	1.122	
	P7	.923	.031	.858	.989	
	P40	.585	.031	.520	.651	
	P42	.619	.031	.554	.685	
	CK	.573	.031	.508	.639	
TPHC	P5	330.000	16.194	296.221	363.779	
	P21	345.000	16.194	311.221	378.779	
	P22	445.000	16.194	411.221	478.779	
	P24	260.000	16.194	226.221	293.779	
	P26	255.000	16.194	221.221	288.779	
	P6	300.000	16.194	266.221	333.779	
	P7	350.000	16.194	316.221	383.779	
	P40	325.000	16.194	291.221	358.779	
	P42	320.000	16.194	286.221	353.779	
	CK	226.000	16.194	192.221	259.779	
APHC	P5	37.067	1.573	33.784	40.349	
	P21	36.533	1.573	33.251	39.816	
	P22	43.500	1.573	40.218	46.782	
	P24	33.700	1.573	30.418	36.982	
	P26	37.283	1.573	34.001	40.566	
	P6	37.350	1.573	34.068	40.632	
	P7	38.750	1.573	35.468	42.032	
	P40	38.250	1.573	34.968	41.532	
	P42	37.300	1.573	34.018	40.582	
	CK	40.800	1.573	37.518	44.082	
TPOC	P5	2.423	.096	2.224	2.623	
	P21	4.560	.096	4.360	4.760	
	P22	3.110	.096	2.910	3.310	
	P24	1.553	.096	1.354	1.753	
	P26	1.320	.096	1.120	1.520	
	P6	1.420	.096	1.220	1.620	
	P7	1.890	.096	1.690	2.090	
	P40	1.995	.096	1.795	2.195	
	P42	2.548	.096	2.349	2.748	
	CK	1.193	.096	.994	1.393	
APOC	P5	.455	.025	.402	.507	
	P21	.338	.025	.285	.390	
	P22	.351	.025	.298	.404	
	P24	.470	.025	.417	.522	
	P26	.409	.025	.356	.461	
	P6	.333	.025	.280	.386	
	P7	.224	.025	.171	.277	
	P40	.297	.025	.244	.349	
	P42	.280	.025	.227	.333	
	CK	.230	.025	.177	.283	
Branchnumber	P5	3.522	.047	3.425	3.620	
	P21	3.862	.047	3.765	3.960	
	P22	3.460	.047	3.362	3.558	
	P24	3.903	.047	3.805	4.001	
	P26	3.000	.047	2.902	3.098	
	P6	3.207	.047	3.109	3.304	
	P7	3.550	.047	3.452	3.648	
	P40	3.640	.047	3.542	3.738	
	P42	4.400	.047	4.302	4.498	
	CK	2.580	.047	2.482	2.678	
rootlength	P5	9.690	.060	9.564	9.816	
	P21	10.250	.060	10.124	10.376	
	P22	13.300	.060	13.174	13.426	
	P24	9.860	.060	9.734	9.986	
	P26	8.380	.060	8.254	8.506	
	P6	11.920	.060	11.794	12.046	
	P7	10.440	.060	10.314	10.566	
	P40	9.800	.060	9.674	9.926	
	P42	11.030	.060	10.904	11.156	
	CK	9.482	.060	9.356	9.607	
plantheight	P5	13.763	.081	13.594	13.932	
	P21	16.840	.081	16.671	17.009	
	P22	16.380	.081	16.211	16.549	
	P24	15.730	.081	15.561	15.899	
	P26	14.160	.081	13.991	14.329	
	P6	15.070	.081	14.901	15.239	
	P7	14.147	.081	13.978	14.316	
	P40	16.140	.081	15.971	16.309	
	P42	16.200	.081	16.031	16.369	
	CK	12.927	.081	12.758	13.096	
chl	P5	1.050	.046	.954	1.146	
	P21	1.220	.046	1.124	1.316	
	P22	1.730	.046	1.634	1.826	
	P24	.736	.046	.640	.831	
	P26	.845	.046	.749	.941	
	P6	1.531	.046	1.436	1.627	
	P7	1.029	.046	.933	1.125	
	P40	1.180	.046	1.084	1.276	
	P42	1.560	.046	1.464	1.656	
	CK	.981	.046	.886	1.077	
leafnumber	P5	30.940	.179	30.567	31.313	
	P21	28.500	.179	28.127	28.873	
	P22	31.300	.179	30.927	31.673	
	P24	28.500	.179	28.127	28.873	
	P26	27.200	.179	26.827	27.573	
	P6	28.500	.179	28.127	28.873	
	P7	24.667	.179	24.293	25.040	
	P40	40.100	.179	39.727	40.473	
	P42	35.070	.179	34.697	35.443	
	CK	27.000	.179	26.627	27.373	


"After this" test


Group


Multiple comparisons	
dependent variable	(I) Group	(J) Group	Mean difference
 (I-J)	Standard error	Sig.	95% confidence interval	
						lower level	Up level	
OCC	LSD	P5	P21	23.333333*	10.1488916	.032	2.163116	44.503550	
			P22	4.666667	10.1488916	.651	-16.503550	25.836884	
			P24	-46.333333*	10.1488916	.000	-67.503550	-25.163116	
			P26	-7.333333	10.1488916	.478	-28.503550	13.836884	
			P6	-35.666667*	10.1488916	.002	-56.836884	-14.496450	
			P7	69.000000*	10.1488916	.000	47.829783	90.170217	
			P40	-50.666667*	10.1488916	.000	-71.836884	-29.496450	
			P42	-23.000000*	10.1488916	.035	-44.170217	-1.829783	
			CK	101.000000*	10.1488916	.000	79.829783	122.170217	
		P21	P5	-23.333333*	10.1488916	.032	-44.503550	-2.163116	
			P22	-18.666667	10.1488916	.081	-39.836884	2.503550	
			P24	-69.666667*	10.1488916	.000	-90.836884	-48.496450	
			P26	-30.666667*	10.1488916	.007	-51.836884	-9.496450	
			P6	-59.000000*	10.1488916	.000	-80.170217	-37.829783	
			P7	45.666667*	10.1488916	.000	24.496450	66.836884	
			P40	-74.000000*	10.1488916	.000	-95.170217	-52.829783	
			P42	-46.333333*	10.1488916	.000	-67.503550	-25.163116	
			CK	77.666667*	10.1488916	.000	56.496450	98.836884	
		P22	P5	-4.666667	10.1488916	.651	-25.836884	16.503550	
			P21	18.666667	10.1488916	.081	-2.503550	39.836884	
			P24	-51.000000*	10.1488916	.000	-72.170217	-29.829783	
			P26	-12.000000	10.1488916	.251	-33.170217	9.170217	
			P6	-40.333333*	10.1488916	.001	-61.503550	-19.163116	
			P7	64.333333*	10.1488916	.000	43.163116	85.503550	
			P40	-55.333333*	10.1488916	.000	-76.503550	-34.163116	
			P42	-27.666667*	10.1488916	.013	-48.836884	-6.496450	
			CK	96.333333*	10.1488916	.000	75.163116	117.503550	
		P24	P5	46.333333*	10.1488916	.000	25.163116	67.503550	
			P21	69.666667*	10.1488916	.000	48.496450	90.836884	
			P22	51.000000*	10.1488916	.000	29.829783	72.170217	
			P26	39.000000*	10.1488916	.001	17.829783	60.170217	
			P6	10.666667	10.1488916	.306	-10.503550	31.836884	
			P7	115.333333*	10.1488916	.000	94.163116	136.503550	
			P40	-4.333333	10.1488916	.674	-25.503550	16.836884	
			P42	23.333333*	10.1488916	.032	2.163116	44.503550	
			CK	147.333333*	10.1488916	.000	126.163116	168.503550	
		P26	P5	7.333333	10.1488916	.478	-13.836884	28.503550	
			P21	30.666667*	10.1488916	.007	9.496450	51.836884	
			P22	12.000000	10.1488916	.251	-9.170217	33.170217	
			P24	-39.000000*	10.1488916	.001	-60.170217	-17.829783	
			P6	-28.333333*	10.1488916	.011	-49.503550	-7.163116	
			P7	76.333333*	10.1488916	.000	55.163116	97.503550	
			P40	-43.333333*	10.1488916	.000	-64.503550	-22.163116	
			P42	-15.666667	10.1488916	.138	-36.836884	5.503550	
			CK	108.333333*	10.1488916	.000	87.163116	129.503550	
		P6	P5	35.666667*	10.1488916	.002	14.496450	56.836884	
			P21	59.000000*	10.1488916	.000	37.829783	80.170217	
			P22	40.333333*	10.1488916	.001	19.163116	61.503550	
			P24	-10.666667	10.1488916	.306	-31.836884	10.503550	
			P26	28.333333*	10.1488916	.011	7.163116	49.503550	
			P7	104.666667*	10.1488916	.000	83.496450	125.836884	
			P40	-15.000000	10.1488916	.155	-36.170217	6.170217	
			P42	12.666667	10.1488916	.226	-8.503550	33.836884	
			CK	136.666667*	10.1488916	.000	115.496450	157.836884	
		P7	P5	-69.000000*	10.1488916	.000	-90.170217	-47.829783	
			P21	-45.666667*	10.1488916	.000	-66.836884	-24.496450	
			P22	-64.333333*	10.1488916	.000	-85.503550	-43.163116	
			P24	-115.333333*	10.1488916	.000	-136.503550	-94.163116	
			P26	-76.333333*	10.1488916	.000	-97.503550	-55.163116	
			P6	-104.666667*	10.1488916	.000	-125.836884	-83.496450	
			P40	-119.666667*	10.1488916	.000	-140.836884	-98.496450	
			P42	-92.000000*	10.1488916	.000	-113.170217	-70.829783	
			CK	32.000000*	10.1488916	.005	10.829783	53.170217	
		P40	P5	50.666667*	10.1488916	.000	29.496450	71.836884	
			P21	74.000000*	10.1488916	.000	52.829783	95.170217	
			P22	55.333333*	10.1488916	.000	34.163116	76.503550	
			P24	4.333333	10.1488916	.674	-16.836884	25.503550	
			P26	43.333333*	10.1488916	.000	22.163116	64.503550	
			P6	15.000000	10.1488916	.155	-6.170217	36.170217	
			P7	119.666667*	10.1488916	.000	98.496450	140.836884	
			P42	27.666667*	10.1488916	.013	6.496450	48.836884	
			CK	151.666667*	10.1488916	.000	130.496450	172.836884	
		P42	P5	23.000000*	10.1488916	.035	1.829783	44.170217	
			P21	46.333333*	10.1488916	.000	25.163116	67.503550	
			P22	27.666667*	10.1488916	.013	6.496450	48.836884	
			P24	-23.333333*	10.1488916	.032	-44.503550	-2.163116	
			P26	15.666667	10.1488916	.138	-5.503550	36.836884	
			P6	-12.666667	10.1488916	.226	-33.836884	8.503550	
			P7	92.000000*	10.1488916	.000	70.829783	113.170217	
			P40	-27.666667*	10.1488916	.013	-48.836884	-6.496450	
			CK	124.000000*	10.1488916	.000	102.829783	145.170217	
		CK	P5	-101.000000*	10.1488916	.000	-122.170217	-79.829783	
			P21	-77.666667*	10.1488916	.000	-98.836884	-56.496450	
			P22	-96.333333*	10.1488916	.000	-117.503550	-75.163116	
			P24	-147.333333*	10.1488916	.000	-168.503550	-126.163116	
			P26	-108.333333*	10.1488916	.000	-129.503550	-87.163116	
			P6	-136.666667*	10.1488916	.000	-157.836884	-115.496450	
			P7	-32.000000*	10.1488916	.005	-53.170217	-10.829783	
			P40	-151.666667*	10.1488916	.000	-172.836884	-130.496450	
			P42	-124.000000*	10.1488916	.000	-145.170217	-102.829783	
TNC	LSD	P5	P21	1.752667	1.4855662	.252	-1.346170	4.851504	
			P22	1.186000	1.4855662	.434	-1.912837	4.284837	
			P24	-1.718333	1.4855662	.261	-4.817170	1.380504	
			P26	-.212000	1.4855662	.888	-3.310837	2.886837	
			P6	-1.404667	1.4855662	.356	-4.503504	1.694170	
			P7	4.224667*	1.4855662	.010	1.125830	7.323504	
			P40	-2.391333	1.4855662	.123	-5.490170	.707504	
			P42	-.557000	1.4855662	.712	-3.655837	2.541837	
			CK	5.452333*	1.4855662	.002	2.353496	8.551170	
		P21	P5	-1.752667	1.4855662	.252	-4.851504	1.346170	
			P22	-.566667	1.4855662	.707	-3.665504	2.532170	
			P24	-3.471000*	1.4855662	.030	-6.569837	-.372163	
			P26	-1.964667	1.4855662	.201	-5.063504	1.134170	
			P6	-3.157333*	1.4855662	.046	-6.256170	-.058496	
			P7	2.472000	1.4855662	.112	-.626837	5.570837	
			P40	-4.144000*	1.4855662	.011	-7.242837	-1.045163	
			P42	-2.309667	1.4855662	.136	-5.408504	.789170	
			CK	3.699667*	1.4855662	.022	.600830	6.798504	
		P22	P5	-1.186000	1.4855662	.434	-4.284837	1.912837	
			P21	.566667	1.4855662	.707	-2.532170	3.665504	
			P24	-2.904333	1.4855662	.065	-6.003170	.194504	
			P26	-1.398000	1.4855662	.358	-4.496837	1.700837	
			P6	-2.590667	1.4855662	.097	-5.689504	.508170	
			P7	3.038667	1.4855662	.054	-.060170	6.137504	
			P40	-3.577333*	1.4855662	.026	-6.676170	-.478496	
			P42	-1.743000	1.4855662	.254	-4.841837	1.355837	
			CK	4.266333*	1.4855662	.009	1.167496	7.365170	
		P24	P5	1.718333	1.4855662	.261	-1.380504	4.817170	
			P21	3.471000*	1.4855662	.030	.372163	6.569837	
			P22	2.904333	1.4855662	.065	-.194504	6.003170	
			P26	1.506333	1.4855662	.323	-1.592504	4.605170	
			P6	.313667	1.4855662	.835	-2.785170	3.412504	
			P7	5.943000*	1.4855662	.001	2.844163	9.041837	
			P40	-.673000	1.4855662	.655	-3.771837	2.425837	
			P42	1.161333	1.4855662	.444	-1.937504	4.260170	
			CK	7.170667*	1.4855662	.000	4.071830	10.269504	
		P26	P5	.212000	1.4855662	.888	-2.886837	3.310837	
			P21	1.964667	1.4855662	.201	-1.134170	5.063504	
			P22	1.398000	1.4855662	.358	-1.700837	4.496837	
			P24	-1.506333	1.4855662	.323	-4.605170	1.592504	
			P6	-1.192667	1.4855662	.432	-4.291504	1.906170	
			P7	4.436667*	1.4855662	.007	1.337830	7.535504	
			P40	-2.179333	1.4855662	.158	-5.278170	.919504	
			P42	-.345000	1.4855662	.819	-3.443837	2.753837	
			CK	5.664333*	1.4855662	.001	2.565496	8.763170	
		P6	P5	1.404667	1.4855662	.356	-1.694170	4.503504	
			P21	3.157333*	1.4855662	.046	.058496	6.256170	
			P22	2.590667	1.4855662	.097	-.508170	5.689504	
			P24	-.313667	1.4855662	.835	-3.412504	2.785170	
			P26	1.192667	1.4855662	.432	-1.906170	4.291504	
			P7	5.629333*	1.4855662	.001	2.530496	8.728170	
			P40	-.986667	1.4855662	.514	-4.085504	2.112170	
			P42	.847667	1.4855662	.575	-2.251170	3.946504	
			CK	6.857000*	1.4855662	.000	3.758163	9.955837	
		P7	P5	-4.224667*	1.4855662	.010	-7.323504	-1.125830	
			P21	-2.472000	1.4855662	.112	-5.570837	.626837	
			P22	-3.038667	1.4855662	.054	-6.137504	.060170	
			P24	-5.943000*	1.4855662	.001	-9.041837	-2.844163	
			P26	-4.436667*	1.4855662	.007	-7.535504	-1.337830	
			P6	-5.629333*	1.4855662	.001	-8.728170	-2.530496	
			P40	-6.616000*	1.4855662	.000	-9.714837	-3.517163	
			P42	-4.781667*	1.4855662	.004	-7.880504	-1.682830	
			CK	1.227667	1.4855662	.418	-1.871170	4.326504	
		P40	P5	2.391333	1.4855662	.123	-.707504	5.490170	
			P21	4.144000*	1.4855662	.011	1.045163	7.242837	
			P22	3.577333*	1.4855662	.026	.478496	6.676170	
			P24	.673000	1.4855662	.655	-2.425837	3.771837	
			P26	2.179333	1.4855662	.158	-.919504	5.278170	
			P6	.986667	1.4855662	.514	-2.112170	4.085504	
			P7	6.616000*	1.4855662	.000	3.517163	9.714837	
			P42	1.834333	1.4855662	.231	-1.264504	4.933170	
			CK	7.843667*	1.4855662	.000	4.744830	10.942504	
		P42	P5	.557000	1.4855662	.712	-2.541837	3.655837	
			P21	2.309667	1.4855662	.136	-.789170	5.408504	
			P22	1.743000	1.4855662	.254	-1.355837	4.841837	
			P24	-1.161333	1.4855662	.444	-4.260170	1.937504	
			P26	.345000	1.4855662	.819	-2.753837	3.443837	
			P6	-.847667	1.4855662	.575	-3.946504	2.251170	
			P7	4.781667*	1.4855662	.004	1.682830	7.880504	
			P40	-1.834333	1.4855662	.231	-4.933170	1.264504	
			CK	6.009333*	1.4855662	.001	2.910496	9.108170	
		CK	P5	-5.452333*	1.4855662	.002	-8.551170	-2.353496	
			P21	-3.699667*	1.4855662	.022	-6.798504	-.600830	
			P22	-4.266333*	1.4855662	.009	-7.365170	-1.167496	
			P24	-7.170667*	1.4855662	.000	-10.269504	-4.071830	
			P26	-5.664333*	1.4855662	.001	-8.763170	-2.565496	
			P6	-6.857000*	1.4855662	.000	-9.955837	-3.758163	
			P7	-1.227667	1.4855662	.418	-4.326504	1.871170	
			P40	-7.843667*	1.4855662	.000	-10.942504	-4.744830	
			P42	-6.009333*	1.4855662	.001	-9.108170	-2.910496	
HNC	LSD	P5	P21	-.080000	.0444512	.087	-.172724	.012724	
			P22	-.160000*	.0444512	.002	-.252724	-.067276	
			P24	.095000*	.0444512	.045	.002276	.187724	
			P26	.142000*	.0444512	.005	.049276	.234724	
			P6	-.276667*	.0444512	.000	-.369390	-.183943	
			P7	-.143333*	.0444512	.004	-.236057	-.050610	
			P40	.194667*	.0444512	.000	.101943	.287390	
			P42	.160667*	.0444512	.002	.067943	.253390	
			CK	.206667*	.0444512	.000	.113943	.299390	
		P21	P5	.080000	.0444512	.087	-.012724	.172724	
			P22	-.080000	.0444512	.087	-.172724	.012724	
			P24	.175000*	.0444512	.001	.082276	.267724	
			P26	.222000*	.0444512	.000	.129276	.314724	
			P6	-.196667*	.0444512	.000	-.289390	-.103943	
			P7	-.063333	.0444512	.170	-.156057	.029390	
			P40	.274667*	.0444512	.000	.181943	.367390	
			P42	.240667*	.0444512	.000	.147943	.333390	
			CK	.286667*	.0444512	.000	.193943	.379390	
		P22	P5	.160000*	.0444512	.002	.067276	.252724	
			P21	.080000	.0444512	.087	-.012724	.172724	
			P24	.255000*	.0444512	.000	.162276	.347724	
			P26	.302000*	.0444512	.000	.209276	.394724	
			P6	-.116667*	.0444512	.016	-.209390	-.023943	
			P7	.016667	.0444512	.712	-.076057	.109390	
			P40	.354667*	.0444512	.000	.261943	.447390	
			P42	.320667*	.0444512	.000	.227943	.413390	
			CK	.366667*	.0444512	.000	.273943	.459390	
		P24	P5	-.095000*	.0444512	.045	-.187724	-.002276	
			P21	-.175000*	.0444512	.001	-.267724	-.082276	
			P22	-.255000*	.0444512	.000	-.347724	-.162276	
			P26	.047000	.0444512	.303	-.045724	.139724	
			P6	-.371667*	.0444512	.000	-.464390	-.278943	
			P7	-.238333*	.0444512	.000	-.331057	-.145610	
			P40	.099667*	.0444512	.036	.006943	.192390	
			P42	.065667	.0444512	.155	-.027057	.158390	
			CK	.111667*	.0444512	.021	.018943	.204390	
		P26	P5	-.142000*	.0444512	.005	-.234724	-.049276	
			P21	-.222000*	.0444512	.000	-.314724	-.129276	
			P22	-.302000*	.0444512	.000	-.394724	-.209276	
			P24	-.047000	.0444512	.303	-.139724	.045724	
			P6	-.418667*	.0444512	.000	-.511390	-.325943	
			P7	-.285333*	.0444512	.000	-.378057	-.192610	
			P40	.052667	.0444512	.250	-.040057	.145390	
			P42	.018667	.0444512	.679	-.074057	.111390	
			CK	.064667	.0444512	.161	-.028057	.157390	
		P6	P5	.276667*	.0444512	.000	.183943	.369390	
			P21	.196667*	.0444512	.000	.103943	.289390	
			P22	.116667*	.0444512	.016	.023943	.209390	
			P24	.371667*	.0444512	.000	.278943	.464390	
			P26	.418667*	.0444512	.000	.325943	.511390	
			P7	.133333*	.0444512	.007	.040610	.226057	
			P40	.471333*	.0444512	.000	.378610	.564057	
			P42	.437333*	.0444512	.000	.344610	.530057	
			CK	.483333*	.0444512	.000	.390610	.576057	
		P7	P5	.143333*	.0444512	.004	.050610	.236057	
			P21	.063333	.0444512	.170	-.029390	.156057	
			P22	-.016667	.0444512	.712	-.109390	.076057	
			P24	.238333*	.0444512	.000	.145610	.331057	
			P26	.285333*	.0444512	.000	.192610	.378057	
			P6	-.133333*	.0444512	.007	-.226057	-.040610	
			P40	.338000*	.0444512	.000	.245276	.430724	
			P42	.304000*	.0444512	.000	.211276	.396724	
			CK	.350000*	.0444512	.000	.257276	.442724	
		P40	P5	-.194667*	.0444512	.000	-.287390	-.101943	
			P21	-.274667*	.0444512	.000	-.367390	-.181943	
			P22	-.354667*	.0444512	.000	-.447390	-.261943	
			P24	-.099667*	.0444512	.036	-.192390	-.006943	
			P26	-.052667	.0444512	.250	-.145390	.040057	
			P6	-.471333*	.0444512	.000	-.564057	-.378610	
			P7	-.338000*	.0444512	.000	-.430724	-.245276	
			P42	-.034000	.0444512	.453	-.126724	.058724	
			CK	.012000	.0444512	.790	-.080724	.104724	
		P42	P5	-.160667*	.0444512	.002	-.253390	-.067943	
			P21	-.240667*	.0444512	.000	-.333390	-.147943	
			P22	-.320667*	.0444512	.000	-.413390	-.227943	
			P24	-.065667	.0444512	.155	-.158390	.027057	
			P26	-.018667	.0444512	.679	-.111390	.074057	
			P6	-.437333*	.0444512	.000	-.530057	-.344610	
			P7	-.304000*	.0444512	.000	-.396724	-.211276	
			P40	.034000	.0444512	.453	-.058724	.126724	
			CK	.046000	.0444512	.313	-.046724	.138724	
		CK	P5	-.206667*	.0444512	.000	-.299390	-.113943	
			P21	-.286667*	.0444512	.000	-.379390	-.193943	
			P22	-.366667*	.0444512	.000	-.459390	-.273943	
			P24	-.111667*	.0444512	.021	-.204390	-.018943	
			P26	-.064667	.0444512	.161	-.157390	.028057	
			P6	-.483333*	.0444512	.000	-.576057	-.390610	
			P7	-.350000*	.0444512	.000	-.442724	-.257276	
			P40	-.012000	.0444512	.790	-.104724	.080724	
			P42	-.046000	.0444512	.313	-.138724	.046724	
TPHC	LSD	P5	P21	-15.000000	22.9012372	.520	-62.771144	32.771144	
			P22	-115.000000*	22.9012372	.000	-162.771144	-67.228856	
			P24	70.000000*	22.9012372	.006	22.228856	117.771144	
			P26	75.000000*	22.9012372	.004	27.228856	122.771144	
			P6	30.000000	22.9012372	.205	-17.771144	77.771144	
			P7	-20.000000	22.9012372	.393	-67.771144	27.771144	
			P40	5.000000	22.9012372	.829	-42.771144	52.771144	
			P42	10.000000	22.9012372	.667	-37.771144	57.771144	
			CK	104.000000*	22.9012372	.000	56.228856	151.771144	
		P21	P5	15.000000	22.9012372	.520	-32.771144	62.771144	
			P22	-100.000000*	22.9012372	.000	-147.771144	-52.228856	
			P24	85.000000*	22.9012372	.001	37.228856	132.771144	
			P26	90.000000*	22.9012372	.001	42.228856	137.771144	
			P6	45.000000	22.9012372	.063	-2.771144	92.771144	
			P7	-5.000000	22.9012372	.829	-52.771144	42.771144	
			P40	20.000000	22.9012372	.393	-27.771144	67.771144	
			P42	25.000000	22.9012372	.288	-22.771144	72.771144	
			CK	119.000000*	22.9012372	.000	71.228856	166.771144	
		P22	P5	115.000000*	22.9012372	.000	67.228856	162.771144	
			P21	100.000000*	22.9012372	.000	52.228856	147.771144	
			P24	185.000000*	22.9012372	.000	137.228856	232.771144	
			P26	190.000000*	22.9012372	.000	142.228856	237.771144	
			P6	145.000000*	22.9012372	.000	97.228856	192.771144	
			P7	95.000000*	22.9012372	.000	47.228856	142.771144	
			P40	120.000000*	22.9012372	.000	72.228856	167.771144	
			P42	125.000000*	22.9012372	.000	77.228856	172.771144	
			CK	219.000000*	22.9012372	.000	171.228856	266.771144	
		P24	P5	-70.000000*	22.9012372	.006	-117.771144	-22.228856	
			P21	-85.000000*	22.9012372	.001	-132.771144	-37.228856	
			P22	-185.000000*	22.9012372	.000	-232.771144	-137.228856	
			P26	5.000000	22.9012372	.829	-42.771144	52.771144	
			P6	-40.000000	22.9012372	.096	-87.771144	7.771144	
			P7	-90.000000*	22.9012372	.001	-137.771144	-42.228856	
			P40	-65.000000*	22.9012372	.010	-112.771144	-17.228856	
			P42	-60.000000*	22.9012372	.016	-107.771144	-12.228856	
			CK	34.000000	22.9012372	.153	-13.771144	81.771144	
		P26	P5	-75.000000*	22.9012372	.004	-122.771144	-27.228856	
			P21	-90.000000*	22.9012372	.001	-137.771144	-42.228856	
			P22	-190.000000*	22.9012372	.000	-237.771144	-142.228856	
			P24	-5.000000	22.9012372	.829	-52.771144	42.771144	
			P6	-45.000000	22.9012372	.063	-92.771144	2.771144	
			P7	-95.000000*	22.9012372	.000	-142.771144	-47.228856	
			P40	-70.000000*	22.9012372	.006	-117.771144	-22.228856	
			P42	-65.000000*	22.9012372	.010	-112.771144	-17.228856	
			CK	29.000000	22.9012372	.220	-18.771144	76.771144	
		P6	P5	-30.000000	22.9012372	.205	-77.771144	17.771144	
			P21	-45.000000	22.9012372	.063	-92.771144	2.771144	
			P22	-145.000000*	22.9012372	.000	-192.771144	-97.228856	
			P24	40.000000	22.9012372	.096	-7.771144	87.771144	
			P26	45.000000	22.9012372	.063	-2.771144	92.771144	
			P7	-50.000000*	22.9012372	.041	-97.771144	-2.228856	
			P40	-25.000000	22.9012372	.288	-72.771144	22.771144	
			P42	-20.000000	22.9012372	.393	-67.771144	27.771144	
			CK	74.000000*	22.9012372	.004	26.228856	121.771144	
		P7	P5	20.000000	22.9012372	.393	-27.771144	67.771144	
			P21	5.000000	22.9012372	.829	-42.771144	52.771144	
			P22	-95.000000*	22.9012372	.000	-142.771144	-47.228856	
			P24	90.000000*	22.9012372	.001	42.228856	137.771144	
			P26	95.000000*	22.9012372	.000	47.228856	142.771144	
			P6	50.000000*	22.9012372	.041	2.228856	97.771144	
			P40	25.000000	22.9012372	.288	-22.771144	72.771144	
			P42	30.000000	22.9012372	.205	-17.771144	77.771144	
			CK	124.000000*	22.9012372	.000	76.228856	171.771144	
		P40	P5	-5.000000	22.9012372	.829	-52.771144	42.771144	
			P21	-20.000000	22.9012372	.393	-67.771144	27.771144	
			P22	-120.000000*	22.9012372	.000	-167.771144	-72.228856	
			P24	65.000000*	22.9012372	.010	17.228856	112.771144	
			P26	70.000000*	22.9012372	.006	22.228856	117.771144	
			P6	25.000000	22.9012372	.288	-22.771144	72.771144	
			P7	-25.000000	22.9012372	.288	-72.771144	22.771144	
			P42	5.000000	22.9012372	.829	-42.771144	52.771144	
			CK	99.000000*	22.9012372	.000	51.228856	146.771144	
		P42	P5	-10.000000	22.9012372	.667	-57.771144	37.771144	
			P21	-25.000000	22.9012372	.288	-72.771144	22.771144	
			P22	-125.000000*	22.9012372	.000	-172.771144	-77.228856	
			P24	60.000000*	22.9012372	.016	12.228856	107.771144	
			P26	65.000000*	22.9012372	.010	17.228856	112.771144	
			P6	20.000000	22.9012372	.393	-27.771144	67.771144	
			P7	-30.000000	22.9012372	.205	-77.771144	17.771144	
			P40	-5.000000	22.9012372	.829	-52.771144	42.771144	
			CK	94.000000*	22.9012372	.001	46.228856	141.771144	
		CK	P5	-104.000000*	22.9012372	.000	-151.771144	-56.228856	
			P21	-119.000000*	22.9012372	.000	-166.771144	-71.228856	
			P22	-219.000000*	22.9012372	.000	-266.771144	-171.228856	
			P24	-34.000000	22.9012372	.153	-81.771144	13.771144	
			P26	-29.000000	22.9012372	.220	-76.771144	18.771144	
			P6	-74.000000*	22.9012372	.004	-121.771144	-26.228856	
			P7	-124.000000*	22.9012372	.000	-171.771144	-76.228856	
			P40	-99.000000*	22.9012372	.000	-146.771144	-51.228856	
			P42	-94.000000*	22.9012372	.001	-141.771144	-46.228856	
APHC	LSD	P5	P21	.533333	2.2252341	.813	-4.108424	5.175090	
			P22	-6.433333*	2.2252341	.009	-11.075090	-1.791576	
			P24	3.366667	2.2252341	.146	-1.275090	8.008424	
			P26	-.216667	2.2252341	.923	-4.858424	4.425090	
			P6	-.283333	2.2252341	.900	-4.925090	4.358424	
			P7	-1.683333	2.2252341	.458	-6.325090	2.958424	
			P40	-1.183333	2.2252341	.601	-5.825090	3.458424	
			P42	-.233333	2.2252341	.918	-4.875090	4.408424	
			CK	-3.733333	2.2252341	.109	-8.375090	.908424	
		P21	P5	-.533333	2.2252341	.813	-5.175090	4.108424	
			P22	-6.966667*	2.2252341	.005	-11.608424	-2.324910	
			P24	2.833333	2.2252341	.218	-1.808424	7.475090	
			P26	-.750000	2.2252341	.740	-5.391757	3.891757	
			P6	-.816667	2.2252341	.717	-5.458424	3.825090	
			P7	-2.216667	2.2252341	.331	-6.858424	2.425090	
			P40	-1.716667	2.2252341	.449	-6.358424	2.925090	
			P42	-.766667	2.2252341	.734	-5.408424	3.875090	
			CK	-4.266667	2.2252341	.070	-8.908424	.375090	
		P22	P5	6.433333*	2.2252341	.009	1.791576	11.075090	
			P21	6.966667*	2.2252341	.005	2.324910	11.608424	
			P24	9.800000*	2.2252341	.000	5.158243	14.441757	
			P26	6.216667*	2.2252341	.011	1.574910	10.858424	
			P6	6.150000*	2.2252341	.012	1.508243	10.791757	
			P7	4.750000*	2.2252341	.045	.108243	9.391757	
			P40	5.250000*	2.2252341	.029	.608243	9.891757	
			P42	6.200000*	2.2252341	.011	1.558243	10.841757	
			CK	2.700000	2.2252341	.239	-1.941757	7.341757	
		P24	P5	-3.366667	2.2252341	.146	-8.008424	1.275090	
			P21	-2.833333	2.2252341	.218	-7.475090	1.808424	
			P22	-9.800000*	2.2252341	.000	-14.441757	-5.158243	
			P26	-3.583333	2.2252341	.123	-8.225090	1.058424	
			P6	-3.650000	2.2252341	.117	-8.291757	.991757	
			P7	-5.050000*	2.2252341	.034	-9.691757	-.408243	
			P40	-4.550000	2.2252341	.054	-9.191757	.091757	
			P42	-3.600000	2.2252341	.121	-8.241757	1.041757	
			CK	-7.100000*	2.2252341	.005	-11.741757	-2.458243	
		P26	P5	.216667	2.2252341	.923	-4.425090	4.858424	
			P21	.750000	2.2252341	.740	-3.891757	5.391757	
			P22	-6.216667*	2.2252341	.011	-10.858424	-1.574910	
			P24	3.583333	2.2252341	.123	-1.058424	8.225090	
			P6	-.066667	2.2252341	.976	-4.708424	4.575090	
			P7	-1.466667	2.2252341	.517	-6.108424	3.175090	
			P40	-.966667	2.2252341	.669	-5.608424	3.675090	
			P42	-.016667	2.2252341	.994	-4.658424	4.625090	
			CK	-3.516667	2.2252341	.130	-8.158424	1.125090	
		P6	P5	.283333	2.2252341	.900	-4.358424	4.925090	
			P21	.816667	2.2252341	.717	-3.825090	5.458424	
			P22	-6.150000*	2.2252341	.012	-10.791757	-1.508243	
			P24	3.650000	2.2252341	.117	-.991757	8.291757	
			P26	.066667	2.2252341	.976	-4.575090	4.708424	
			P7	-1.400000	2.2252341	.536	-6.041757	3.241757	
			P40	-.900000	2.2252341	.690	-5.541757	3.741757	
			P42	.050000	2.2252341	.982	-4.591757	4.691757	
			CK	-3.450000	2.2252341	.137	-8.091757	1.191757	
		P7	P5	1.683333	2.2252341	.458	-2.958424	6.325090	
			P21	2.216667	2.2252341	.331	-2.425090	6.858424	
			P22	-4.750000*	2.2252341	.045	-9.391757	-.108243	
			P24	5.050000*	2.2252341	.034	.408243	9.691757	
			P26	1.466667	2.2252341	.517	-3.175090	6.108424	
			P6	1.400000	2.2252341	.536	-3.241757	6.041757	
			P40	.500000	2.2252341	.824	-4.141757	5.141757	
			P42	1.450000	2.2252341	.522	-3.191757	6.091757	
			CK	-2.050000	2.2252341	.368	-6.691757	2.591757	
		P40	P5	1.183333	2.2252341	.601	-3.458424	5.825090	
			P21	1.716667	2.2252341	.449	-2.925090	6.358424	
			P22	-5.250000*	2.2252341	.029	-9.891757	-.608243	
			P24	4.550000	2.2252341	.054	-.091757	9.191757	
			P26	.966667	2.2252341	.669	-3.675090	5.608424	
			P6	.900000	2.2252341	.690	-3.741757	5.541757	
			P7	-.500000	2.2252341	.824	-5.141757	4.141757	
			P42	.950000	2.2252341	.674	-3.691757	5.591757	
			CK	-2.550000	2.2252341	.265	-7.191757	2.091757	
		P42	P5	.233333	2.2252341	.918	-4.408424	4.875090	
			P21	.766667	2.2252341	.734	-3.875090	5.408424	
			P22	-6.200000*	2.2252341	.011	-10.841757	-1.558243	
			P24	3.600000	2.2252341	.121	-1.041757	8.241757	
			P26	.016667	2.2252341	.994	-4.625090	4.658424	
			P6	-.050000	2.2252341	.982	-4.691757	4.591757	
			P7	-1.450000	2.2252341	.522	-6.091757	3.191757	
			P40	-.950000	2.2252341	.674	-5.591757	3.691757	
			CK	-3.500000	2.2252341	.131	-8.141757	1.141757	
		CK	P5	3.733333	2.2252341	.109	-.908424	8.375090	
			P21	4.266667	2.2252341	.070	-.375090	8.908424	
			P22	-2.700000	2.2252341	.239	-7.341757	1.941757	
			P24	7.100000*	2.2252341	.005	2.458243	11.741757	
			P26	3.516667	2.2252341	.130	-1.125090	8.158424	
			P6	3.450000	2.2252341	.137	-1.191757	8.091757	
			P7	2.050000	2.2252341	.368	-2.591757	6.691757	
			P40	2.550000	2.2252341	.265	-2.091757	7.191757	
			P42	3.500000	2.2252341	.131	-1.141757	8.141757	
TPOC	LSD	P5	P21	-2.136667*	.1353473	.000	-2.418996	-1.854337	
			P22	-.686667*	.1353473	.000	-.968996	-.404337	
			P24	.870000*	.1353473	.000	.587671	1.152329	
			P26	1.103333*	.1353473	.000	.821004	1.385663	
			P6	1.003333*	.1353473	.000	.721004	1.285663	
			P7	.533333*	.1353473	.001	.251004	.815663	
			P40	.428333*	.1353473	.005	.146004	.710663	
			P42	-.125000	.1353473	.367	-.407329	.157329	
			CK	1.230000*	.1353473	.000	.947671	1.512329	
		P21	P5	2.136667*	.1353473	.000	1.854337	2.418996	
			P22	1.450000*	.1353473	.000	1.167671	1.732329	
			P24	3.006667*	.1353473	.000	2.724337	3.288996	
			P26	3.240000*	.1353473	.000	2.957671	3.522329	
			P6	3.140000*	.1353473	.000	2.857671	3.422329	
			P7	2.670000*	.1353473	.000	2.387671	2.952329	
			P40	2.565000*	.1353473	.000	2.282671	2.847329	
			P42	2.011667*	.1353473	.000	1.729337	2.293996	
			CK	3.366667*	.1353473	.000	3.084337	3.648996	
		P22	P5	.686667*	.1353473	.000	.404337	.968996	
			P21	-1.450000*	.1353473	.000	-1.732329	-1.167671	
			P24	1.556667*	.1353473	.000	1.274337	1.838996	
			P26	1.790000*	.1353473	.000	1.507671	2.072329	
			P6	1.690000*	.1353473	.000	1.407671	1.972329	
			P7	1.220000*	.1353473	.000	.937671	1.502329	
			P40	1.115000*	.1353473	.000	.832671	1.397329	
			P42	.561667*	.1353473	.000	.279337	.843996	
			CK	1.916667*	.1353473	.000	1.634337	2.198996	
		P24	P5	-.870000*	.1353473	.000	-1.152329	-.587671	
			P21	-3.006667*	.1353473	.000	-3.288996	-2.724337	
			P22	-1.556667*	.1353473	.000	-1.838996	-1.274337	
			P26	.233333	.1353473	.100	-.048996	.515663	
			P6	.133333	.1353473	.336	-.148996	.415663	
			P7	-.336667*	.1353473	.022	-.618996	-.054337	
			P40	-.441667*	.1353473	.004	-.723996	-.159337	
			P42	-.995000*	.1353473	.000	-1.277329	-.712671	
			CK	.360000*	.1353473	.015	.077671	.642329	
		P26	P5	-1.103333*	.1353473	.000	-1.385663	-.821004	
			P21	-3.240000*	.1353473	.000	-3.522329	-2.957671	
			P22	-1.790000*	.1353473	.000	-2.072329	-1.507671	
			P24	-.233333	.1353473	.100	-.515663	.048996	
			P6	-.100000	.1353473	.469	-.382329	.182329	
			P7	-.570000*	.1353473	.000	-.852329	-.287671	
			P40	-.675000*	.1353473	.000	-.957329	-.392671	
			P42	-1.228333*	.1353473	.000	-1.510663	-.946004	
			CK	.126667	.1353473	.361	-.155663	.408996	
		P6	P5	-1.003333*	.1353473	.000	-1.285663	-.721004	
			P21	-3.140000*	.1353473	.000	-3.422329	-2.857671	
			P22	-1.690000*	.1353473	.000	-1.972329	-1.407671	
			P24	-.133333	.1353473	.336	-.415663	.148996	
			P26	.100000	.1353473	.469	-.182329	.382329	
			P7	-.470000*	.1353473	.002	-.752329	-.187671	
			P40	-.575000*	.1353473	.000	-.857329	-.292671	
			P42	-1.128333*	.1353473	.000	-1.410663	-.846004	
			CK	.226667	.1353473	.110	-.055663	.508996	
		P7	P5	-.533333*	.1353473	.001	-.815663	-.251004	
			P21	-2.670000*	.1353473	.000	-2.952329	-2.387671	
			P22	-1.220000*	.1353473	.000	-1.502329	-.937671	
			P24	.336667*	.1353473	.022	.054337	.618996	
			P26	.570000*	.1353473	.000	.287671	.852329	
			P6	.470000*	.1353473	.002	.187671	.752329	
			P40	-.105000	.1353473	.447	-.387329	.177329	
			P42	-.658333*	.1353473	.000	-.940663	-.376004	
			CK	.696667*	.1353473	.000	.414337	.978996	
		P40	P5	-.428333*	.1353473	.005	-.710663	-.146004	
			P21	-2.565000*	.1353473	.000	-2.847329	-2.282671	
			P22	-1.115000*	.1353473	.000	-1.397329	-.832671	
			P24	.441667*	.1353473	.004	.159337	.723996	
			P26	.675000*	.1353473	.000	.392671	.957329	
			P6	.575000*	.1353473	.000	.292671	.857329	
			P7	.105000	.1353473	.447	-.177329	.387329	
			P42	-.553333*	.1353473	.001	-.835663	-.271004	
			CK	.801667*	.1353473	.000	.519337	1.083996	
		P42	P5	.125000	.1353473	.367	-.157329	.407329	
			P21	-2.011667*	.1353473	.000	-2.293996	-1.729337	
			P22	-.561667*	.1353473	.000	-.843996	-.279337	
			P24	.995000*	.1353473	.000	.712671	1.277329	
			P26	1.228333*	.1353473	.000	.946004	1.510663	
			P6	1.128333*	.1353473	.000	.846004	1.410663	
			P7	.658333*	.1353473	.000	.376004	.940663	
			P40	.553333*	.1353473	.001	.271004	.835663	
			CK	1.355000*	.1353473	.000	1.072671	1.637329	
		CK	P5	-1.230000*	.1353473	.000	-1.512329	-.947671	
			P21	-3.366667*	.1353473	.000	-3.648996	-3.084337	
			P22	-1.916667*	.1353473	.000	-2.198996	-1.634337	
			P24	-.360000*	.1353473	.015	-.642329	-.077671	
			P26	-.126667	.1353473	.361	-.408996	.155663	
			P6	-.226667	.1353473	.110	-.508996	.055663	
			P7	-.696667*	.1353473	.000	-.978996	-.414337	
			P40	-.801667*	.1353473	.000	-1.083996	-.519337	
			P42	-1.355000*	.1353473	.000	-1.637329	-1.072671	
APOC	LSD	P5	P21	.116967*	.0355961	.004	.042714	.191219	
			P22	.103500*	.0355961	.009	.029248	.177752	
			P24	-.015000	.0355961	.678	-.089252	.059252	
			P26	.046000	.0355961	.211	-.028252	.120252	
			P6	.121500*	.0355961	.003	.047248	.195752	
			P7	.230500*	.0355961	.000	.156248	.304752	
			P40	.158000*	.0355961	.000	.083748	.232252	
			P42	.174500*	.0355961	.000	.100248	.248752	
			CK	.224500*	.0355961	.000	.150248	.298752	
		P21	P5	-.116967*	.0355961	.004	-.191219	-.042714	
			P22	-.013467	.0355961	.709	-.087719	.060786	
			P24	-.131967*	.0355961	.001	-.206219	-.057714	
			P26	-.070967	.0355961	.060	-.145219	.003286	
			P6	.004533	.0355961	.900	-.069719	.078786	
			P7	.113533*	.0355961	.005	.039281	.187786	
			P40	.041033	.0355961	.263	-.033219	.115286	
			P42	.057533	.0355961	.122	-.016719	.131786	
			CK	.107533*	.0355961	.007	.033281	.181786	
		P22	P5	-.103500*	.0355961	.009	-.177752	-.029248	
			P21	.013467	.0355961	.709	-.060786	.087719	
			P24	-.118500*	.0355961	.003	-.192752	-.044248	
			P26	-.057500	.0355961	.122	-.131752	.016752	
			P6	.018000	.0355961	.619	-.056252	.092252	
			P7	.127000*	.0355961	.002	.052748	.201252	
			P40	.054500	.0355961	.141	-.019752	.128752	
			P42	.071000	.0355961	.060	-.003252	.145252	
			CK	.121000*	.0355961	.003	.046748	.195252	
		P24	P5	.015000	.0355961	.678	-.059252	.089252	
			P21	.131967*	.0355961	.001	.057714	.206219	
			P22	.118500*	.0355961	.003	.044248	.192752	
			P26	.061000	.0355961	.102	-.013252	.135252	
			P6	.136500*	.0355961	.001	.062248	.210752	
			P7	.245500*	.0355961	.000	.171248	.319752	
			P40	.173000*	.0355961	.000	.098748	.247252	
			P42	.189500*	.0355961	.000	.115248	.263752	
			CK	.239500*	.0355961	.000	.165248	.313752	
		P26	P5	-.046000	.0355961	.211	-.120252	.028252	
			P21	.070967	.0355961	.060	-.003286	.145219	
			P22	.057500	.0355961	.122	-.016752	.131752	
			P24	-.061000	.0355961	.102	-.135252	.013252	
			P6	.075500*	.0355961	.047	.001248	.149752	
			P7	.184500*	.0355961	.000	.110248	.258752	
			P40	.112000*	.0355961	.005	.037748	.186252	
			P42	.128500*	.0355961	.002	.054248	.202752	
			CK	.178500*	.0355961	.000	.104248	.252752	
		P6	P5	-.121500*	.0355961	.003	-.195752	-.047248	
			P21	-.004533	.0355961	.900	-.078786	.069719	
			P22	-.018000	.0355961	.619	-.092252	.056252	
			P24	-.136500*	.0355961	.001	-.210752	-.062248	
			P26	-.075500*	.0355961	.047	-.149752	-.001248	
			P7	.109000*	.0355961	.006	.034748	.183252	
			P40	.036500	.0355961	.317	-.037752	.110752	
			P42	.053000	.0355961	.152	-.021252	.127252	
			CK	.103000*	.0355961	.009	.028748	.177252	
		P7	P5	-.230500*	.0355961	.000	-.304752	-.156248	
			P21	-.113533*	.0355961	.005	-.187786	-.039281	
			P22	-.127000*	.0355961	.002	-.201252	-.052748	
			P24	-.245500*	.0355961	.000	-.319752	-.171248	
			P26	-.184500*	.0355961	.000	-.258752	-.110248	
			P6	-.109000*	.0355961	.006	-.183252	-.034748	
			P40	-.072500	.0355961	.055	-.146752	.001752	
			P42	-.056000	.0355961	.131	-.130252	.018252	
			CK	-.006000	.0355961	.868	-.080252	.068252	
		P40	P5	-.158000*	.0355961	.000	-.232252	-.083748	
			P21	-.041033	.0355961	.263	-.115286	.033219	
			P22	-.054500	.0355961	.141	-.128752	.019752	
			P24	-.173000*	.0355961	.000	-.247252	-.098748	
			P26	-.112000*	.0355961	.005	-.186252	-.037748	
			P6	-.036500	.0355961	.317	-.110752	.037752	
			P7	.072500	.0355961	.055	-.001752	.146752	
			P42	.016500	.0355961	.648	-.057752	.090752	
			CK	.066500	.0355961	.076	-.007752	.140752	
		P42	P5	-.174500*	.0355961	.000	-.248752	-.100248	
			P21	-.057533	.0355961	.122	-.131786	.016719	
			P22	-.071000	.0355961	.060	-.145252	.003252	
			P24	-.189500*	.0355961	.000	-.263752	-.115248	
			P26	-.128500*	.0355961	.002	-.202752	-.054248	
			P6	-.053000	.0355961	.152	-.127252	.021252	
			P7	.056000	.0355961	.131	-.018252	.130252	
			P40	-.016500	.0355961	.648	-.090752	.057752	
			CK	.050000	.0355961	.175	-.024252	.124252	
		CK	P5	-.224500*	.0355961	.000	-.298752	-.150248	
			P21	-.107533*	.0355961	.007	-.181786	-.033281	
			P22	-.121000*	.0355961	.003	-.195252	-.046748	
			P24	-.239500*	.0355961	.000	-.313752	-.165248	
			P26	-.178500*	.0355961	.000	-.252752	-.104248	
			P6	-.103000*	.0355961	.009	-.177252	-.028748	
			P7	.006000	.0355961	.868	-.068252	.080252	
			P40	-.066500	.0355961	.076	-.140752	.007752	
			P42	-.050000	.0355961	.175	-.124252	.024252	
Branchnumber	LSD	P5	P21	-.339967*	.0662258	.000	-.478111	-.201822	
			P22	.062233	.0662258	.359	-.075911	.200378	
			P24	-.380767*	.0662258	.000	-.518911	-.242622	
			P26	.522233*	.0662258	.000	.384089	.660378	
			P6	.315567*	.0662258	.000	.177422	.453711	
			P7	-.027767	.0662258	.679	-.165911	.110378	
			P40	-.117767	.0662258	.091	-.255911	.020378	
			P42	-.877767*	.0662258	.000	-1.015911	-.739622	
			CK	.942233*	.0662258	.000	.804089	1.080378	
		P21	P5	.339967*	.0662258	.000	.201822	.478111	
			P22	.402200*	.0662258	.000	.264055	.540345	
			P24	-.040800	.0662258	.545	-.178945	.097345	
			P26	.862200*	.0662258	.000	.724055	1.000345	
			P6	.655533*	.0662258	.000	.517389	.793678	
			P7	.312200*	.0662258	.000	.174055	.450345	
			P40	.222200*	.0662258	.003	.084055	.360345	
			P42	-.537800*	.0662258	.000	-.675945	-.399655	
			CK	1.282200*	.0662258	.000	1.144055	1.420345	
		P22	P5	-.062233	.0662258	.359	-.200378	.075911	
			P21	-.402200*	.0662258	.000	-.540345	-.264055	
			P24	-.443000*	.0662258	.000	-.581145	-.304855	
			P26	.460000*	.0662258	.000	.321855	.598145	
			P6	.253333*	.0662258	.001	.115189	.391478	
			P7	-.090000	.0662258	.189	-.228145	.048145	
			P40	-.180000*	.0662258	.013	-.318145	-.041855	
			P42	-.940000*	.0662258	.000	-1.078145	-.801855	
			CK	.880000*	.0662258	.000	.741855	1.018145	
		P24	P5	.380767*	.0662258	.000	.242622	.518911	
			P21	.040800	.0662258	.545	-.097345	.178945	
			P22	.443000*	.0662258	.000	.304855	.581145	
			P26	.903000*	.0662258	.000	.764855	1.041145	
			P6	.696333*	.0662258	.000	.558189	.834478	
			P7	.353000*	.0662258	.000	.214855	.491145	
			P40	.263000*	.0662258	.001	.124855	.401145	
			P42	-.497000*	.0662258	.000	-.635145	-.358855	
			CK	1.323000*	.0662258	.000	1.184855	1.461145	
		P26	P5	-.522233*	.0662258	.000	-.660378	-.384089	
			P21	-.862200*	.0662258	.000	-1.000345	-.724055	
			P22	-.460000*	.0662258	.000	-.598145	-.321855	
			P24	-.903000*	.0662258	.000	-1.041145	-.764855	
			P6	-.206667*	.0662258	.005	-.344811	-.068522	
			P7	-.550000*	.0662258	.000	-.688145	-.411855	
			P40	-.640000*	.0662258	.000	-.778145	-.501855	
			P42	-1.400000*	.0662258	.000	-1.538145	-1.261855	
			CK	.420000*	.0662258	.000	.281855	.558145	
		P6	P5	-.315567*	.0662258	.000	-.453711	-.177422	
			P21	-.655533*	.0662258	.000	-.793678	-.517389	
			P22	-.253333*	.0662258	.001	-.391478	-.115189	
			P24	-.696333*	.0662258	.000	-.834478	-.558189	
			P26	.206667*	.0662258	.005	.068522	.344811	
			P7	-.343333*	.0662258	.000	-.481478	-.205189	
			P40	-.433333*	.0662258	.000	-.571478	-.295189	
			P42	-1.193333*	.0662258	.000	-1.331478	-1.055189	
			CK	.626667*	.0662258	.000	.488522	.764811	
		P7	P5	.027767	.0662258	.679	-.110378	.165911	
			P21	-.312200*	.0662258	.000	-.450345	-.174055	
			P22	.090000	.0662258	.189	-.048145	.228145	
			P24	-.353000*	.0662258	.000	-.491145	-.214855	
			P26	.550000*	.0662258	.000	.411855	.688145	
			P6	.343333*	.0662258	.000	.205189	.481478	
			P40	-.090000	.0662258	.189	-.228145	.048145	
			P42	-.850000*	.0662258	.000	-.988145	-.711855	
			CK	.970000*	.0662258	.000	.831855	1.108145	
		P40	P5	.117767	.0662258	.091	-.020378	.255911	
			P21	-.222200*	.0662258	.003	-.360345	-.084055	
			P22	.180000*	.0662258	.013	.041855	.318145	
			P24	-.263000*	.0662258	.001	-.401145	-.124855	
			P26	.640000*	.0662258	.000	.501855	.778145	
			P6	.433333*	.0662258	.000	.295189	.571478	
			P7	.090000	.0662258	.189	-.048145	.228145	
			P42	-.760000*	.0662258	.000	-.898145	-.621855	
			CK	1.060000*	.0662258	.000	.921855	1.198145	
		P42	P5	.877767*	.0662258	.000	.739622	1.015911	
			P21	.537800*	.0662258	.000	.399655	.675945	
			P22	.940000*	.0662258	.000	.801855	1.078145	
			P24	.497000*	.0662258	.000	.358855	.635145	
			P26	1.400000*	.0662258	.000	1.261855	1.538145	
			P6	1.193333*	.0662258	.000	1.055189	1.331478	
			P7	.850000*	.0662258	.000	.711855	.988145	
			P40	.760000*	.0662258	.000	.621855	.898145	
			CK	1.820000*	.0662258	.000	1.681855	1.958145	
		CK	P5	-.942233*	.0662258	.000	-1.080378	-.804089	
			P21	-1.282200*	.0662258	.000	-1.420345	-1.144055	
			P22	-.880000*	.0662258	.000	-1.018145	-.741855	
			P24	-1.323000*	.0662258	.000	-1.461145	-1.184855	
			P26	-.420000*	.0662258	.000	-.558145	-.281855	
			P6	-.626667*	.0662258	.000	-.764811	-.488522	
			P7	-.970000*	.0662258	.000	-1.108145	-.831855	
			P40	-1.060000*	.0662258	.000	-1.198145	-.921855	
			P42	-1.820000*	.0662258	.000	-1.958145	-1.681855	
rootlength	LSD	P5	P21	-.560000*	.0851502	.000	-.737620	-.382380	
			P22	-3.610000*	.0851502	.000	-3.787620	-3.432380	
			P24	-.170000	.0851502	.060	-.347620	.007620	
			P26	1.310000*	.0851502	.000	1.132380	1.487620	
			P6	-2.230000*	.0851502	.000	-2.407620	-2.052380	
			P7	-.750000*	.0851502	.000	-.927620	-.572380	
			P40	-.110000	.0851502	.211	-.287620	.067620	
			P42	-1.340000*	.0851502	.000	-1.517620	-1.162380	
			CK	.208333*	.0851502	.024	.030713	.385954	
		P21	P5	.560000*	.0851502	.000	.382380	.737620	
			P22	-3.050000*	.0851502	.000	-3.227620	-2.872380	
			P24	.390000*	.0851502	.000	.212380	.567620	
			P26	1.870000*	.0851502	.000	1.692380	2.047620	
			P6	-1.670000*	.0851502	.000	-1.847620	-1.492380	
			P7	-.190000*	.0851502	.037	-.367620	-.012380	
			P40	.450000*	.0851502	.000	.272380	.627620	
			P42	-.780000*	.0851502	.000	-.957620	-.602380	
			CK	.768333*	.0851502	.000	.590713	.945954	
		P22	P5	3.610000*	.0851502	.000	3.432380	3.787620	
			P21	3.050000*	.0851502	.000	2.872380	3.227620	
			P24	3.440000*	.0851502	.000	3.262380	3.617620	
			P26	4.920000*	.0851502	.000	4.742380	5.097620	
			P6	1.380000*	.0851502	.000	1.202380	1.557620	
			P7	2.860000*	.0851502	.000	2.682380	3.037620	
			P40	3.500000*	.0851502	.000	3.322380	3.677620	
			P42	2.270000*	.0851502	.000	2.092380	2.447620	
			CK	3.818333*	.0851502	.000	3.640713	3.995954	
		P24	P5	.170000	.0851502	.060	-.007620	.347620	
			P21	-.390000*	.0851502	.000	-.567620	-.212380	
			P22	-3.440000*	.0851502	.000	-3.617620	-3.262380	
			P26	1.480000*	.0851502	.000	1.302380	1.657620	
			P6	-2.060000*	.0851502	.000	-2.237620	-1.882380	
			P7	-.580000*	.0851502	.000	-.757620	-.402380	
			P40	.060000	.0851502	.489	-.117620	.237620	
			P42	-1.170000*	.0851502	.000	-1.347620	-.992380	
			CK	.378333*	.0851502	.000	.200713	.555954	
		P26	P5	-1.310000*	.0851502	.000	-1.487620	-1.132380	
			P21	-1.870000*	.0851502	.000	-2.047620	-1.692380	
			P22	-4.920000*	.0851502	.000	-5.097620	-4.742380	
			P24	-1.480000*	.0851502	.000	-1.657620	-1.302380	
			P6	-3.540000*	.0851502	.000	-3.717620	-3.362380	
			P7	-2.060000*	.0851502	.000	-2.237620	-1.882380	
			P40	-1.420000*	.0851502	.000	-1.597620	-1.242380	
			P42	-2.650000*	.0851502	.000	-2.827620	-2.472380	
			CK	-1.101667*	.0851502	.000	-1.279287	-.924046	
		P6	P5	2.230000*	.0851502	.000	2.052380	2.407620	
			P21	1.670000*	.0851502	.000	1.492380	1.847620	
			P22	-1.380000*	.0851502	.000	-1.557620	-1.202380	
			P24	2.060000*	.0851502	.000	1.882380	2.237620	
			P26	3.540000*	.0851502	.000	3.362380	3.717620	
			P7	1.480000*	.0851502	.000	1.302380	1.657620	
			P40	2.120000*	.0851502	.000	1.942380	2.297620	
			P42	.890000*	.0851502	.000	.712380	1.067620	
			CK	2.438333*	.0851502	.000	2.260713	2.615954	
		P7	P5	.750000*	.0851502	.000	.572380	.927620	
			P21	.190000*	.0851502	.037	.012380	.367620	
			P22	-2.860000*	.0851502	.000	-3.037620	-2.682380	
			P24	.580000*	.0851502	.000	.402380	.757620	
			P26	2.060000*	.0851502	.000	1.882380	2.237620	
			P6	-1.480000*	.0851502	.000	-1.657620	-1.302380	
			P40	.640000*	.0851502	.000	.462380	.817620	
			P42	-.590000*	.0851502	.000	-.767620	-.412380	
			CK	.958333*	.0851502	.000	.780713	1.135954	
		P40	P5	.110000	.0851502	.211	-.067620	.287620	
			P21	-.450000*	.0851502	.000	-.627620	-.272380	
			P22	-3.500000*	.0851502	.000	-3.677620	-3.322380	
			P24	-.060000	.0851502	.489	-.237620	.117620	
			P26	1.420000*	.0851502	.000	1.242380	1.597620	
			P6	-2.120000*	.0851502	.000	-2.297620	-1.942380	
			P7	-.640000*	.0851502	.000	-.817620	-.462380	
			P42	-1.230000*	.0851502	.000	-1.407620	-1.052380	
			CK	.318333*	.0851502	.001	.140713	.495954	
		P42	P5	1.340000*	.0851502	.000	1.162380	1.517620	
			P21	.780000*	.0851502	.000	.602380	.957620	
			P22	-2.270000*	.0851502	.000	-2.447620	-2.092380	
			P24	1.170000*	.0851502	.000	.992380	1.347620	
			P26	2.650000*	.0851502	.000	2.472380	2.827620	
			P6	-.890000*	.0851502	.000	-1.067620	-.712380	
			P7	.590000*	.0851502	.000	.412380	.767620	
			P40	1.230000*	.0851502	.000	1.052380	1.407620	
			CK	1.548333*	.0851502	.000	1.370713	1.725954	
		CK	P5	-.208333*	.0851502	.024	-.385954	-.030713	
			P21	-.768333*	.0851502	.000	-.945954	-.590713	
			P22	-3.818333*	.0851502	.000	-3.995954	-3.640713	
			P24	-.378333*	.0851502	.000	-.555954	-.200713	
			P26	1.101667*	.0851502	.000	.924046	1.279287	
			P6	-2.438333*	.0851502	.000	-2.615954	-2.260713	
			P7	-.958333*	.0851502	.000	-1.135954	-.780713	
			P40	-.318333*	.0851502	.001	-.495954	-.140713	
			P42	-1.548333*	.0851502	.000	-1.725954	-1.370713	
plantheight	LSD	P5	P21	-3.0767*	.11454	.000	-3.3156	-2.8377	
			P22	-2.6167*	.11454	.000	-2.8556	-2.3777	
			P24	-1.9667*	.11454	.000	-2.2056	-1.7277	
			P26	-.3967*	.11454	.002	-.6356	-.1577	
			P6	-1.3067*	.11454	.000	-1.5456	-1.0677	
			P7	-.3833*	.11454	.003	-.6223	-.1444	
			P40	-2.3767*	.11454	.000	-2.6156	-2.1377	
			P42	-2.4367*	.11454	.000	-2.6756	-2.1977	
			CK	.8367*	.11454	.000	.5977	1.0756	
		P21	P5	3.0767*	.11454	.000	2.8377	3.3156	
			P22	.4600*	.11454	.001	.2211	.6989	
			P24	1.1100*	.11454	.000	.8711	1.3489	
			P26	2.6800*	.11454	.000	2.4411	2.9189	
			P6	1.7700*	.11454	.000	1.5311	2.0089	
			P7	2.6933*	.11454	.000	2.4544	2.9323	
			P40	.7000*	.11454	.000	.4611	.9389	
			P42	.6400*	.11454	.000	.4011	.8789	
			CK	3.9133*	.11454	.000	3.6744	4.1523	
		P22	P5	2.6167*	.11454	.000	2.3777	2.8556	
			P21	-.4600*	.11454	.001	-.6989	-.2211	
			P24	.6500*	.11454	.000	.4111	.8889	
			P26	2.2200*	.11454	.000	1.9811	2.4589	
			P6	1.3100*	.11454	.000	1.0711	1.5489	
			P7	2.2333*	.11454	.000	1.9944	2.4723	
			P40	.2400*	.11454	.049	.0011	.4789	
			P42	.1800	.11454	.132	-.0589	.4189	
			CK	3.4533*	.11454	.000	3.2144	3.6923	
		P24	P5	1.9667*	.11454	.000	1.7277	2.2056	
			P21	-1.1100*	.11454	.000	-1.3489	-.8711	
			P22	-.6500*	.11454	.000	-.8889	-.4111	
			P26	1.5700*	.11454	.000	1.3311	1.8089	
			P6	.6600*	.11454	.000	.4211	.8989	
			P7	1.5833*	.11454	.000	1.3444	1.8223	
			P40	-.4100*	.11454	.002	-.6489	-.1711	
			P42	-.4700*	.11454	.001	-.7089	-.2311	
			CK	2.8033*	.11454	.000	2.5644	3.0423	
		P26	P5	.3967*	.11454	.002	.1577	.6356	
			P21	-2.6800*	.11454	.000	-2.9189	-2.4411	
			P22	-2.2200*	.11454	.000	-2.4589	-1.9811	
			P24	-1.5700*	.11454	.000	-1.8089	-1.3311	
			P6	-.9100*	.11454	.000	-1.1489	-.6711	
			P7	.0133	.11454	.908	-.2256	.2523	
			P40	-1.9800*	.11454	.000	-2.2189	-1.7411	
			P42	-2.0400*	.11454	.000	-2.2789	-1.8011	
			CK	1.2333*	.11454	.000	.9944	1.4723	
		P6	P5	1.3067*	.11454	.000	1.0677	1.5456	
			P21	-1.7700*	.11454	.000	-2.0089	-1.5311	
			P22	-1.3100*	.11454	.000	-1.5489	-1.0711	
			P24	-.6600*	.11454	.000	-.8989	-.4211	
			P26	.9100*	.11454	.000	.6711	1.1489	
			P7	.9233*	.11454	.000	.6844	1.1623	
			P40	-1.0700*	.11454	.000	-1.3089	-.8311	
			P42	-1.1300*	.11454	.000	-1.3689	-.8911	
			CK	2.1433*	.11454	.000	1.9044	2.3823	
		P7	P5	.3833*	.11454	.003	.1444	.6223	
			P21	-2.6933*	.11454	.000	-2.9323	-2.4544	
			P22	-2.2333*	.11454	.000	-2.4723	-1.9944	
			P24	-1.5833*	.11454	.000	-1.8223	-1.3444	
			P26	-.0133	.11454	.908	-.2523	.2256	
			P6	-.9233*	.11454	.000	-1.1623	-.6844	
			P40	-1.9933*	.11454	.000	-2.2323	-1.7544	
			P42	-2.0533*	.11454	.000	-2.2923	-1.8144	
			CK	1.2200*	.11454	.000	.9811	1.4589	
		P40	P5	2.3767*	.11454	.000	2.1377	2.6156	
			P21	-.7000*	.11454	.000	-.9389	-.4611	
			P22	-.2400*	.11454	.049	-.4789	-.0011	
			P24	.4100*	.11454	.002	.1711	.6489	
			P26	1.9800*	.11454	.000	1.7411	2.2189	
			P6	1.0700*	.11454	.000	.8311	1.3089	
			P7	1.9933*	.11454	.000	1.7544	2.2323	
			P42	-.0600	.11454	.606	-.2989	.1789	
			CK	3.2133*	.11454	.000	2.9744	3.4523	
		P42	P5	2.4367*	.11454	.000	2.1977	2.6756	
			P21	-.6400*	.11454	.000	-.8789	-.4011	
			P22	-.1800	.11454	.132	-.4189	.0589	
			P24	.4700*	.11454	.001	.2311	.7089	
			P26	2.0400*	.11454	.000	1.8011	2.2789	
			P6	1.1300*	.11454	.000	.8911	1.3689	
			P7	2.0533*	.11454	.000	1.8144	2.2923	
			P40	.0600	.11454	.606	-.1789	.2989	
			CK	3.2733*	.11454	.000	3.0344	3.5123	
		CK	P5	-.8367*	.11454	.000	-1.0756	-.5977	
			P21	-3.9133*	.11454	.000	-4.1523	-3.6744	
			P22	-3.4533*	.11454	.000	-3.6923	-3.2144	
			P24	-2.8033*	.11454	.000	-3.0423	-2.5644	
			P26	-1.2333*	.11454	.000	-1.4723	-.9944	
			P6	-2.1433*	.11454	.000	-2.3823	-1.9044	
			P7	-1.2200*	.11454	.000	-1.4589	-.9811	
			P40	-3.2133*	.11454	.000	-3.4523	-2.9744	
			P42	-3.2733*	.11454	.000	-3.5123	-3.0344	
chl	LSD	P5	P21	-.170000*	.0648896	.016	-.305357	-.034643	
			P22	-.680000*	.0648896	.000	-.815357	-.544643	
			P24	.314333*	.0648896	.000	.178976	.449691	
			P26	.205000*	.0648896	.005	.069643	.340357	
			P6	-.481333*	.0648896	.000	-.616691	-.345976	
			P7	.021000	.0648896	.750	-.114357	.156357	
			P40	-.130000	.0648896	.059	-.265357	.005357	
			P42	-.510000*	.0648896	.000	-.645357	-.374643	
			CK	.068667	.0648896	.303	-.066691	.204024	
		P21	P5	.170000*	.0648896	.016	.034643	.305357	
			P22	-.510000*	.0648896	.000	-.645357	-.374643	
			P24	.484333*	.0648896	.000	.348976	.619691	
			P26	.375000*	.0648896	.000	.239643	.510357	
			P6	-.311333*	.0648896	.000	-.446691	-.175976	
			P7	.191000*	.0648896	.008	.055643	.326357	
			P40	.040000	.0648896	.545	-.095357	.175357	
			P42	-.340000*	.0648896	.000	-.475357	-.204643	
			CK	.238667*	.0648896	.001	.103309	.374024	
		P22	P5	.680000*	.0648896	.000	.544643	.815357	
			P21	.510000*	.0648896	.000	.374643	.645357	
			P24	.994333*	.0648896	.000	.858976	1.129691	
			P26	.885000*	.0648896	.000	.749643	1.020357	
			P6	.198667*	.0648896	.006	.063309	.334024	
			P7	.701000*	.0648896	.000	.565643	.836357	
			P40	.550000*	.0648896	.000	.414643	.685357	
			P42	.170000*	.0648896	.016	.034643	.305357	
			CK	.748667*	.0648896	.000	.613309	.884024	
		P24	P5	-.314333*	.0648896	.000	-.449691	-.178976	
			P21	-.484333*	.0648896	.000	-.619691	-.348976	
			P22	-.994333*	.0648896	.000	-1.129691	-.858976	
			P26	-.109333	.0648896	.108	-.244691	.026024	
			P6	-.795667*	.0648896	.000	-.931024	-.660309	
			P7	-.293333*	.0648896	.000	-.428691	-.157976	
			P40	-.444333*	.0648896	.000	-.579691	-.308976	
			P42	-.824333*	.0648896	.000	-.959691	-.688976	
			CK	-.245667*	.0648896	.001	-.381024	-.110309	
		P26	P5	-.205000*	.0648896	.005	-.340357	-.069643	
			P21	-.375000*	.0648896	.000	-.510357	-.239643	
			P22	-.885000*	.0648896	.000	-1.020357	-.749643	
			P24	.109333	.0648896	.108	-.026024	.244691	
			P6	-.686333*	.0648896	.000	-.821691	-.550976	
			P7	-.184000*	.0648896	.010	-.319357	-.048643	
			P40	-.335000*	.0648896	.000	-.470357	-.199643	
			P42	-.715000*	.0648896	.000	-.850357	-.579643	
			CK	-.136333*	.0648896	.049	-.271691	-.000976	
		P6	P5	.481333*	.0648896	.000	.345976	.616691	
			P21	.311333*	.0648896	.000	.175976	.446691	
			P22	-.198667*	.0648896	.006	-.334024	-.063309	
			P24	.795667*	.0648896	.000	.660309	.931024	
			P26	.686333*	.0648896	.000	.550976	.821691	
			P7	.502333*	.0648896	.000	.366976	.637691	
			P40	.351333*	.0648896	.000	.215976	.486691	
			P42	-.028667	.0648896	.663	-.164024	.106691	
			CK	.550000*	.0648896	.000	.414643	.685357	
		P7	P5	-.021000	.0648896	.750	-.156357	.114357	
			P21	-.191000*	.0648896	.008	-.326357	-.055643	
			P22	-.701000*	.0648896	.000	-.836357	-.565643	
			P24	.293333*	.0648896	.000	.157976	.428691	
			P26	.184000*	.0648896	.010	.048643	.319357	
			P6	-.502333*	.0648896	.000	-.637691	-.366976	
			P40	-.151000*	.0648896	.031	-.286357	-.015643	
			P42	-.531000*	.0648896	.000	-.666357	-.395643	
			CK	.047667	.0648896	.471	-.087691	.183024	
		P40	P5	.130000	.0648896	.059	-.005357	.265357	
			P21	-.040000	.0648896	.545	-.175357	.095357	
			P22	-.550000*	.0648896	.000	-.685357	-.414643	
			P24	.444333*	.0648896	.000	.308976	.579691	
			P26	.335000*	.0648896	.000	.199643	.470357	
			P6	-.351333*	.0648896	.000	-.486691	-.215976	
			P7	.151000*	.0648896	.031	.015643	.286357	
			P42	-.380000*	.0648896	.000	-.515357	-.244643	
			CK	.198667*	.0648896	.006	.063309	.334024	
		P42	P5	.510000*	.0648896	.000	.374643	.645357	
			P21	.340000*	.0648896	.000	.204643	.475357	
			P22	-.170000*	.0648896	.016	-.305357	-.034643	
			P24	.824333*	.0648896	.000	.688976	.959691	
			P26	.715000*	.0648896	.000	.579643	.850357	
			P6	.028667	.0648896	.663	-.106691	.164024	
			P7	.531000*	.0648896	.000	.395643	.666357	
			P40	.380000*	.0648896	.000	.244643	.515357	
			CK	.578667*	.0648896	.000	.443309	.714024	
		CK	P5	-.068667	.0648896	.303	-.204024	.066691	
			P21	-.238667*	.0648896	.001	-.374024	-.103309	
			P22	-.748667*	.0648896	.000	-.884024	-.613309	
			P24	.245667*	.0648896	.001	.110309	.381024	
			P26	.136333*	.0648896	.049	.000976	.271691	
			P6	-.550000*	.0648896	.000	-.685357	-.414643	
			P7	-.047667	.0648896	.471	-.183024	.087691	
			P40	-.198667*	.0648896	.006	-.334024	-.063309	
			P42	-.578667*	.0648896	.000	-.714024	-.443309	
leafnumber	LSD	P5	P21	2.440000*	.2531183	.000	1.912004	2.967996	
			P22	-.360000	.2531183	.170	-.887996	.167996	
			P24	2.440000*	.2531183	.000	1.912004	2.967996	
			P26	3.740000*	.2531183	.000	3.212004	4.267996	
			P6	2.440000*	.2531183	.000	1.912004	2.967996	
			P7	6.273333*	.2531183	.000	5.745338	6.801329	
			P40	-9.160000*	.2531183	.000	-9.687996	-8.632004	
			P42	-4.130000*	.2531183	.000	-4.657996	-3.602004	
			CK	3.940000*	.2531183	.000	3.412004	4.467996	
		P21	P5	-2.440000*	.2531183	.000	-2.967996	-1.912004	
			P22	-2.800000*	.2531183	.000	-3.327996	-2.272004	
			P24	.000000	.2531183	1.000	-.527996	.527996	
			P26	1.300000*	.2531183	.000	.772004	1.827996	
			P6	.000000	.2531183	1.000	-.527996	.527996	
			P7	3.833333*	.2531183	.000	3.305338	4.361329	
			P40	-11.600000*	.2531183	.000	-12.127996	-11.072004	
			P42	-6.570000*	.2531183	.000	-7.097996	-6.042004	
			CK	1.500000*	.2531183	.000	.972004	2.027996	
		P22	P5	.360000	.2531183	.170	-.167996	.887996	
			P21	2.800000*	.2531183	.000	2.272004	3.327996	
			P24	2.800000*	.2531183	.000	2.272004	3.327996	
			P26	4.100000*	.2531183	.000	3.572004	4.627996	
			P6	2.800000*	.2531183	.000	2.272004	3.327996	
			P7	6.633333*	.2531183	.000	6.105338	7.161329	
			P40	-8.800000*	.2531183	.000	-9.327996	-8.272004	
			P42	-3.770000*	.2531183	.000	-4.297996	-3.242004	
			CK	4.300000*	.2531183	.000	3.772004	4.827996	
		P24	P5	-2.440000*	.2531183	.000	-2.967996	-1.912004	
			P21	.000000	.2531183	1.000	-.527996	.527996	
			P22	-2.800000*	.2531183	.000	-3.327996	-2.272004	
			P26	1.300000*	.2531183	.000	.772004	1.827996	
			P6	.000000	.2531183	1.000	-.527996	.527996	
			P7	3.833333*	.2531183	.000	3.305338	4.361329	
			P40	-11.600000*	.2531183	.000	-12.127996	-11.072004	
			P42	-6.570000*	.2531183	.000	-7.097996	-6.042004	
			CK	1.500000*	.2531183	.000	.972004	2.027996	
		P26	P5	-3.740000*	.2531183	.000	-4.267996	-3.212004	
			P21	-1.300000*	.2531183	.000	-1.827996	-.772004	
			P22	-4.100000*	.2531183	.000	-4.627996	-3.572004	
			P24	-1.300000*	.2531183	.000	-1.827996	-.772004	
			P6	-1.300000*	.2531183	.000	-1.827996	-.772004	
			P7	2.533333*	.2531183	.000	2.005338	3.061329	
			P40	-12.900000*	.2531183	.000	-13.427996	-12.372004	
			P42	-7.870000*	.2531183	.000	-8.397996	-7.342004	
			CK	.200000	.2531183	.439	-.327996	.727996	
		P6	P5	-2.440000*	.2531183	.000	-2.967996	-1.912004	
			P21	.000000	.2531183	1.000	-.527996	.527996	
			P22	-2.800000*	.2531183	.000	-3.327996	-2.272004	
			P24	.000000	.2531183	1.000	-.527996	.527996	
			P26	1.300000*	.2531183	.000	.772004	1.827996	
			P7	3.833333*	.2531183	.000	3.305338	4.361329	
			P40	-11.600000*	.2531183	.000	-12.127996	-11.072004	
			P42	-6.570000*	.2531183	.000	-7.097996	-6.042004	
			CK	1.500000*	.2531183	.000	.972004	2.027996	
		P7	P5	-6.273333*	.2531183	.000	-6.801329	-5.745338	
			P21	-3.833333*	.2531183	.000	-4.361329	-3.305338	
			P22	-6.633333*	.2531183	.000	-7.161329	-6.105338	
			P24	-3.833333*	.2531183	.000	-4.361329	-3.305338	
			P26	-2.533333*	.2531183	.000	-3.061329	-2.005338	
			P6	-3.833333*	.2531183	.000	-4.361329	-3.305338	
			P40	-15.433333*	.2531183	.000	-15.961329	-14.905338	
			P42	-10.403333*	.2531183	.000	-10.931329	-9.875338	
			CK	-2.333333*	.2531183	.000	-2.861329	-1.805338	
		P40	P5	9.160000*	.2531183	.000	8.632004	9.687996	
			P21	11.600000*	.2531183	.000	11.072004	12.127996	
			P22	8.800000*	.2531183	.000	8.272004	9.327996	
			P24	11.600000*	.2531183	.000	11.072004	12.127996	
			P26	12.900000*	.2531183	.000	12.372004	13.427996	
			P6	11.600000*	.2531183	.000	11.072004	12.127996	
			P7	15.433333*	.2531183	.000	14.905338	15.961329	
			P42	5.030000*	.2531183	.000	4.502004	5.557996	
			CK	13.100000*	.2531183	.000	12.572004	13.627996	
		P42	P5	4.130000*	.2531183	.000	3.602004	4.657996	
			P21	6.570000*	.2531183	.000	6.042004	7.097996	
			P22	3.770000*	.2531183	.000	3.242004	4.297996	
			P24	6.570000*	.2531183	.000	6.042004	7.097996	
			P26	7.870000*	.2531183	.000	7.342004	8.397996	
			P6	6.570000*	.2531183	.000	6.042004	7.097996	
			P7	10.403333*	.2531183	.000	9.875338	10.931329	
			P40	-5.030000*	.2531183	.000	-5.557996	-4.502004	
			CK	8.070000*	.2531183	.000	7.542004	8.597996	
		CK	P5	-3.940000*	.2531183	.000	-4.467996	-3.412004	
			P21	-1.500000*	.2531183	.000	-2.027996	-.972004	
			P22	-4.300000*	.2531183	.000	-4.827996	-3.772004	
			P24	-1.500000*	.2531183	.000	-2.027996	-.972004	
			P26	-.200000	.2531183	.439	-.727996	.327996	
			P6	-1.500000*	.2531183	.000	-2.027996	-.972004	
			P7	2.333333*	.2531183	.000	1.805338	2.861329	
			P40	-13.100000*	.2531183	.000	-13.627996	-12.572004	
			P42	-8.070000*	.2531183	.000	-8.597996	-7.542004	

Based on the observed mean.
The error term is mean squared (error) =.096.	
*.The mean difference is more significant at.05 level.	


Homogeneous subset

OCC	
	Group	N	Subset	
			1	2	3	4	5	
Duncana,b,c	CK	3	433.000000					
	P7	3		465.000000				
	P21	3			510.666667			
	P22	3			529.333333	529.333333		
	P5	3				534.000000		
	P26	3				541.333333	541.333333	
	P42	3					557.000000	
	P6	3						
	P24	3						
	P40	3						
	Sig.		1.000	1.000	.081	.277	.138	

OCC	
	Group	Subset	
		6	7	
Duncana,b,c	CK			
	P7			
	P21			
	P22			
	P5			
	P26			
	P42	557.000000		
	P6	569.666667	569.666667	
	P24		580.333333	
	P40		584.666667	
	Sig.	.226	.177	

Means for groups in homogeneous subsets are displayed.	
a. Uses Harmonic Mean Sample Size = 3.000¡£	
b. The group sizes are not equal. A harmonic mean of group sizes will be used. Type I error level is not guaranteed	
c. Alpha = .05。	


TNC	
	Group	N	Subset	
			1	2	3	4	
Duncana,b,c	CK	3	21.331667				
	P7	3	22.559333	22.559333			
	P21	3		25.031333	25.031333		
	P22	3		25.598000	25.598000		
	P5	3			26.784000	26.784000	
	P26	3			26.996000	26.996000	
	P42	3			27.341000	27.341000	
	P6	3			28.188667	28.188667	
	P24	3			28.502333	28.502333	
	P40	3				29.175333	
	Sig.		.418	.066	.052	.168	

Means for groups in homogeneous subsets are displayed.	
a. Uses Harmonic Mean Sample Size = 3.000¡£	
b. The group sizes are not equal. A harmonic mean of group sizes will be used. Type I error level is not guaranteed	
c. Alpha = .05。	


HNC	
	Group	N	Subset	
			1	2	3	4	5	
Duncana,b,c	CK	3	.573333					
	P40	3	.585333	.585333				
	P42	3	.619333	.619333				
	P26	3	.638000	.638000				
	P24	3		.685000				
	P5	3			.780000			
	P21	3			.860000	.860000		
	P7	3				.923333		
	P22	3				.940000		
	P6	3					1.056667	
	Sig.		.197	.051	.087	.103	1.000	

Means for groups in homogeneous subsets are displayed.	
a. Uses Harmonic Mean Sample Size = 3.000¡£	
b. The group sizes are not equal. A harmonic mean of group sizes will be used. Type I error level is not guaranteed	
c. Alpha = .05。	


TPHC	
	Group	N	Subset	
			1	2	3	4	
Duncana,b,c	CK	3	226.000000				
	P26	3	255.000000	255.000000			
	P24	3	260.000000	260.000000			
	P6	3		300.000000	300.000000		
	P42	3			320.000000		
	P40	3			325.000000		
	P5	3			330.000000		
	P21	3			345.000000		
	P7	3			350.000000		
	P22	3				445.000000	
	Sig.		.175	.076	.066	1.000	

Means for groups in homogeneous subsets are displayed.	
a. Uses Harmonic Mean Sample Size = 3.000¡£	
b. The group sizes are not equal. A harmonic mean of group sizes will be used. Type I error level is not guaranteed	
c. Alpha = .05。	


APHC	
	Group	N	Subset	
			1	2	3	
Duncana,b,c	P24	3	33.700000			
	P21	3	36.533333	36.533333		
	P5	3	37.066667	37.066667		
	P26	3	37.283333	37.283333		
	P42	3	37.300000	37.300000		
	P6	3	37.350000	37.350000		
	P40	3	38.250000	38.250000		
	P7	3	38.750000	38.750000	38.750000	
	CK	3		40.800000	40.800000	
	P22	3			43.500000	
	Sig.		.061	.109	.056	

Means for groups in homogeneous subsets are displayed.	
a. Uses Harmonic Mean Sample Size = 3.000¡£	
b. The group sizes are not equal. A harmonic mean of group sizes will be used. Type I error level is not guaranteed	
c. Alpha = .05。	


TPOC	
	Group	N	Subset	
			1	2	3	4	5	6	
Duncana,b,c	CK	3	1.193333						
	P26	3	1.320000	1.320000					
	P6	3	1.420000	1.420000					
	P24	3		1.553333					
	P7	3			1.890000				
	P40	3			1.995000				
	P5	3				2.423333			
	P42	3				2.548333			
	P22	3					3.110000		
	P21	3						4.560000	
	Sig.		.128	.117	.447	.367	1.000	1.000	

Means for groups in homogeneous subsets are displayed.	
a. Uses Harmonic Mean Sample Size = 3.000¡£	
b. The group sizes are not equal. A harmonic mean of group sizes will be used. Type I error level is not guaranteed	
c. Alpha = .05。	


APOC	
	Group	N	Subset	
			1	2	3	4	
Duncana,b,c	P7	3	.224000				
	CK	3	.230000				
	P42	3	.280000	.280000			
	P40	3	.296500	.296500			
	P6	3		.333000	.333000		
	P21	3		.337533	.337533		
	P22	3		.351000	.351000		
	P26	3			.408500	.408500	
	P5	3				.454500	
	P24	3				.469500	
	Sig.		.075	.087	.064	.120	

Means for groups in homogeneous subsets are displayed.	
a. Uses Harmonic Mean Sample Size = 3.000¡£	
b. The group sizes are not equal. A harmonic mean of group sizes will be used. Type I error level is not guaranteed	
c. Alpha = .05。	


Branchnumber	
	Group	N	Subset	
			1	2	3	4	5	6	
Duncana,b,c	CK	3	2.580000						
	P26	3		3.000000					
	P6	3			3.206667				
	P22	3				3.460000			
	P5	3				3.522233	3.522233		
	P7	3				3.550000	3.550000		
	P40	3					3.640000		
	P21	3						3.862200	
	P24	3						3.903000	
	P42	3							
	Sig.		1.000	1.000	1.000	.213	.107	.545	

Branchnumber	
	Group	Subset	
		7	
Duncana,b,c	CK		
	P26		
	P6		
	P22		
	P5		
	P7		
	P40		
	P21		
	P24		
	P42	4.400000	
	Sig.	1.000	

Means for groups in homogeneous subsets are displayed.	
a. Uses Harmonic Mean Sample Size = 3.000¡£	
b. The group sizes are not equal. A harmonic mean of group sizes will be used. Type I error level is not guaranteed	
c. Alpha = .05。	


rootlength	
	Group	N	Subset	
			1	2	3	4	5	
Duncana,b,c	P26	3	8.380000					
	CK	3		9.481667				
	P5	3			9.690000			
	P40	3			9.800000			
	P24	3			9.860000			
	P21	3				10.250000		
	P7	3					10.440000	
	P42	3						
	P6	3						
	P22	3						
	Sig.		1.000	1.000	.072	1.000	1.000	

rootlength	
	Group	Subset	
		6	7	8	
Duncana,b,c	P26				
	CK				
	P5				
	P40				
	P24				
	P21				
	P7				
	P42	11.030000			
	P6		11.920000		
	P22			13.300000	
	Sig.	1.000	1.000	1.000	

Means for groups in homogeneous subsets are displayed.	
a. Uses Harmonic Mean Sample Size = 3.000¡£	
b. The group sizes are not equal. A harmonic mean of group sizes will be used. Type I error level is not guaranteed	
c. Alpha = .05。	


plantheight	
	Group	N	Subset	
			1	2	3	4	5	6	
Duncana,b,c	CK	3	12.9267						
	P5	3		13.7633					
	P7	3			14.1467				
	P26	3			14.1600				
	P6	3				15.0700			
	P24	3					15.7300		
	P40	3						16.1400	
	P42	3						16.2000	
	P22	3						16.3800	
	P21	3							
	Sig.		1.000	1.000	.908	1.000	1.000	.060	

plantheight	
	Group	Subset	
		7	
Duncana,b,c	CK		
	P5		
	P7		
	P26		
	P6		
	P24		
	P40		
	P42		
	P22		
	P21	16.8400	
	Sig.	1.000	

Means for groups in homogeneous subsets are displayed.	
a. Uses Harmonic Mean Sample Size = 3.000¡£	
b. The group sizes are not equal. A harmonic mean of group sizes will be used. Type I error level is not guaranteed	
c. Alpha = .05。	


chl	
	Group	N	Subset	
			1	2	3	4	5	6	
Duncana,b,c	P24	3	.735667						
	P26	3	.845000						
	CK	3		.981333					
	P7	3		1.029000					
	P5	3		1.050000	1.050000				
	P40	3			1.180000	1.180000			
	P21	3				1.220000			
	P6	3					1.531333		
	P42	3					1.560000		
	P22	3						1.730000	
	Sig.		.108	.329	.059	.545	.663	1.000	

Means for groups in homogeneous subsets are displayed.	
a. Uses Harmonic Mean Sample Size = 3.000¡£	
b. The group sizes are not equal. A harmonic mean of group sizes will be used. Type I error level is not guaranteed	
c. Alpha = .05。	


leafnumber	
	Group	N	Subset	
			1	2	3	4	5	
Duncana,b,c	P7	3	24.666667					
	CK	3		27.000000				
	P26	3		27.200000				
	P21	3			28.500000			
	P24	3			28.500000			
	P6	3			28.500000			
	P5	3				30.940000		
	P22	3				31.300000		
	P42	3					35.070000	
	P40	3						
	Sig.		1.000	.439	1.000	.170	1.000	

leafnumber	
	Group	Subset	
		6	
Duncana,b,c	P7		
	CK		
	P26		
	P21		
	P24		
	P6		
	P5		
	P22		
	P42		
	P40	40.100000	
	Sig.	1.000	

Means for groups in homogeneous subsets are displayed.	
a. Uses Harmonic Mean Sample Size = 3.000¡£	
b. The group sizes are not equal. A harmonic mean of group sizes will be used. Type I error level is not guaranteed	
c. Alpha = .05。	
